# Diagnostic value of radiomics in predicting Ki-67 and cytokeratin 19 expression in hepatocellular carcinoma: a systematic review and meta-analysis

**DOI:** 10.3389/fonc.2023.1323534

**Published:** 2024-01-03

**Authors:** Lu Zhou, Yiheng Chen, Yan Li, Chaoyong Wu, Chongxiang Xue, Xihong Wang

**Affiliations:** ^1^ Traditional Chinese Medicine (Zhong Jing) School, Henan University of Chinese Medicine, Zhengzhou, Henan, China; ^2^ Shenzhen Hospital of Beijing University of Chinese Medicine, Shenzhen, China; ^3^ Graduate School, Beijing University of Chinese Medicine, Beijing, China; ^4^ The First Affiliated Hospital of Henan University of Chinese Medicine, Zhengzhou, Henan, China

**Keywords:** radiomics, hepatocellular carcinoma, Ki-67, CK-19, C-index

## Abstract

**Background:**

Radiomics have been increasingly used in the clinical management of hepatocellular carcinoma (HCC), such as markers prediction. Ki-67 and cytokeratin 19 (CK-19) are important prognostic markers of HCC. Radiomics has been introduced by many researchers in the prediction of these markers expression, but its diagnostic value remains controversial. Therefore, this review aims to assess the diagnostic value of radiomics in predicting Ki-67 and CK-19 expression in HCC.

**Methods:**

Original studies were systematically searched in PubMed, EMBASE, Cochrane Library, and Web of Science from inception to May 2023. All included studies were evaluated by the radiomics quality score. The C-index was used as the effect size of the performance of radiomics in predicting Ki-67and CK-19 expression, and the positive cutoff values of Ki-67 label index (LI) were determined by subgroup analysis and meta-regression.

**Results:**

We identified 34 eligible studies for Ki-67 (18 studies) and CK-19 (16 studies). The most common radiomics source was magnetic resonance imaging (MRI; 25/34). The pooled C-index of MRI-based models in predicting Ki-67 was 0.89 (95% CI:0.86–0.92) in the training set, and 0.87 (95% CI: 0.82–0.92) in the validation set. The pooled C-index of MRI-based models in predicting CK-19 was 0.86 (95% CI:0.81–0.90) in the training set, and 0.79 (95% CI: 0.73–0.84) in the validation set. Subgroup analysis suggested Ki-67 LI cutoff was a significant source of heterogeneity (*I* ^2^ = 0.0% *P>*0.05), and meta-regression showed that the C-index increased as Ki-67 LI increased.

**Conclusion:**

Radiomics shows promising diagnostic value in predicting positive Ki-67 or CK-19 expression. But lacks standardized guidelines, which makes the model and variables selection dependent on researcher experience, leading to study heterogeneity. Therefore, standardized guidelines are warranted for future research.

**Systematic Review Registration:**

https://www.crd.york.ac.uk/PROSPERO/, identifier CRD42023427953.

## 1 Introduction

Hepatocellular carcinoma (HCC) is the most common primary liver malignancy. HCC ranks sixth in cancer incidence and third in cancer mortality, and has a 5-year survival rate of <20% (1, 2). Surgical resection is currently the preferred treatment for HCC (3). Unfortunately, HCC patients have poor prognosis, and the 5-year recurrence rate following liver resection ranges from 50% to 70% (4). Therefore, the identification of prognostic factors for HCC is important for developing and improving HCC treatments and prognosis.

Several factors such as microvascular invasion (MVI), tumor grade, Ki-67 and CK-19 expression are closely associated with HCC prognosis (5–7). Ki-67 is a cell proliferation marker present in all active stages of cell cycle and can reflect HCC progression. Positive Ki-67 expression often implies a biologically invasive phenotype in HCC as well as poor overall survival (8, 9). In addition, a study further found that Ki-67 protein expression level is an independent predictor for tumor growth rate and poor prognosis in HCC (10). For the patients with high Ki-67 expression, adjuvant hepatic arterial chemoembolization has been shown to decrease the risk of tumor recurrence after liver tumor resection and prolong the overall survival (11). In addition, systematic chemotherapy based on transarterial chemoembolization are also effective treatments for HCC (12). CK-19 is a cytoskeletal protein mainly found in epithelial cells, such as hepatocytes, biliary epithelial cells, and intrahepatic bile duct cells. High CK-19 expression is significantly associated with poor survival and early tumor recurrence in HCC patients (13). Furthermore, CK-19 was found to be valuable for distinguishing HCC from extrahepatic metastatic tumors (14). Nonetheless, CK-19 positive HCC patients were shown to be more likely to develop drug resistance and fail chemotherapy (15). And further study showed that patients with positive CK-19 expression can benefit from regorafenib, which facilitates the development of personalized therapy for HCC (16). Hence, CK-19 is not only a prognostic marker but also a potential therapeutic target for HCC. Therefore, Ki-67 and CK-19 expression levels in HCC are important for determining the course of disease, prognosis and treatment options.

Based on the above, accurate measurement of Ki-67 and CK-19 expression status helps to guide surveillance and adjuvant treatment strategies. At present, Ki-67 or CK-19 expression is primarily measured by immunohistochemistry. Nevertheless, tumor tissue biopsies are subject to sampling errors and false-negative results, and the procedure increases the risks of complications such as subcapsular hemorrhage and needle tract metastases.

Radiomics is a medical imaging technique that extracts quantitative imaging features from medical images with the help of computer software and selects valuable features by statistical and/or machine learning methods for the analysis of disease characterization, efficacy evaluation and prognosis prediction (17). In cancer research, radiomics has been shown to be useful in identifying tumor pathology information in medical imaging, such as tumor grade, progression and gene expression, without the need of tumor biopsy, thus allowing non-invasive detection of tumor pathology at multiple time points (18). Therefore, radiomics in combination with dynamic detection of Ki-67 and CK-19 expression is useful for timely assessment of HCC progression, and has important clinical implications in early HCC intervention and expansion of the treatment window for patients.

In recent years, numerous studies have constructed radiomics models for predicting Ki-67 or CK19 status based on magnetic resonance imaging (MRI), computed tomography (CT) or ultrasound (US) images. However, the diagnostic accuracy of these radiomics models has been inconsistent. Therefore, this systematic review and meta-analysis was performed to assess the accuracy of radiomics in predicting Ki-67 and CK-19 expression in HCC.

## 2 Methods

### 2.1 Study registration

This study was conducted in accordance with the Preferred Reporting Item for Systematic Reviews and Meta-Analysis (PRISMA 2020) and was registered on PROSPERO (CRD42023427953).

### 2.2 Eligibility criteria

#### 2.2.1 Inclusion criteria

(1) Studies that used radiomics for predicting Ki-67 or CK-19 expression in HCC.
(2) HCC was diagnosed by any recognized diagnostic criteria.
(3) Studies that provided a clear description of Ki-67 or CK-19 expression status.
(4) Cohort, observational, prospective, retrospective, multi-center or case-control studies that assessed the diagnostic value of radiomics in predicting Ki-67 and CK-19 expression in HCC.

#### 2.2.2 Exclusion criteria

(1) Studies with controversial diagnostic criteria for HCC.
(2) Studies that lacked a clear description of Ki-67 or CK-19 expression status.
(3) Review, meta-analysis, case report, expert comment, letter, and conference abstract.
(4) Only comparative analysis of radiomics features, but no diagnostic model was constructed.
(5) Diagnostics models with missing outcome measures (e.g., ROC, C-statistic, C-index, sensitivity, specificity, accuracy, precision, recall, F1 score, confusion matrix, diagnostic contingency table).
(6) Image segmentation and reconstruction were performed, but the diagnostic value of segmentation or reconstruction for predicting high Ki-67 or CK-19 expression was not reported.

### 2.3 Data sources and search strategy

Relevant studies were systematically searched in PubMed, Web of Science, Embase, and Cochrane Library from inception to May 10, 2023 using the MeSH and entry terms “radiomics,” “Ki-67,” “CK-19”, and “hepatocellular carcinoma”. The search strategy is shown in **Supplementary Table 1**. The reference lists of included studies were also manually searched to identify any relevant articles.

### 2.4 Study selection and data extraction

All retrieved studies were imported into Endnote 20, and duplicates were removed. After screening the titles and abstracts, the full texts of potentially eligible studies were downloaded for further assessment. Data were extracted into a standard data extraction table, including first author, country, year of publication, model, number of patients, radiomics source (e.g., MRI, CT, PET), feature source (e.g., radiomics or radiomics and clinical risk factors), and model (e.g., logistic regression). In addition, C-index or area under the receiver operating characteristic curve (AUROC), sensitivity, specificity, and accuracy were also extracted for data processing and forest plot generation. C-index was the primary outcome measure of this systematic review.

Study selection and data extraction were completed by two independent researchers, and any disagreement was resolved by a third researcher.

### 2.5 Quality assessment

Risk of bias and methodological quality were examined by two independent researchers according to the radiomics quality score (RQS) (18). The RQS is a standardized evaluation and reporting guideline that minimizes bias and enhances the usefulness of radiomics-based prediction models. This scale has 16 key components and a maximum score of 36 points. A higher QRS indicates higher quality. Discrepancies in quality score assessment were resolved by re-assessment and discussion.

### 2.6 Outcomes

The primary outcome measures of this study included the C-index of radiomics in the prediction of Ki-67 and CK-19 expression, as well as sensitivity and specificity. C-index is the probability that a correct diagnosis of Ki-67 or CK-19 coincides with the actual observed results. Moreover, in medical diagnosis, sensitivity and specificity are also important metrics for assessing the accuracy of diagnostic methods. Sensitivity refers to the ability of the test method to correctly determine the presence of a disease. Specificity refers to the ability of a test method to correctly determine that the patient does not have a certain disease. Improving sensitivity and specificity can help avoid missed diagnosis and misdiagnosis, respectively.

### 2.7 Synthesis methods

In the meta-analysis of C-index, the *I* ^2^ statistic was used to measure the variation across studies due to heterogeneity rather than sampling error, and is expressed as a percentage of total variation. When *I* ^2^> 50%, which indicates statistical heterogeneity, a random effects model was used for analysis; otherwise, a fixed effects model was used. In addition, the meta-analysis of sensitivity and specificity was performed using a bivariate mixed effects model based on the diagnostic contingency table. The diagnostic contingency table was estimated using sensitivity, specificity, precision, accuracy, number of positive cases, and total number of cases. All analyses were performed using Stata SE version 15.

### 2.8 Data cleaning and preprocessing

For data cleaning and preprocessing, C-index, sensitivity, specificity, and confidence interval (CI) values were first unified with all decimals retained. Missing C-index value, 95% CI or standard error was imputed according to the Debray methods (19). When only the ROC was provided in the original study, sensitivity and specificity were extracted under different probability cutoff values using Origin 2021 and summed to generate the diagnostic contingency table.

### 2.9 Subgroup analysis

Subgroup analysis was performed to identify potential influencing factors, including the positive Ki-67 threshold, internal and external validation, and use of radiomics in combination with clinical risk factors.

Since different positive Ki-67 thresholds have been reported in the original studies, a subgroup analysis was needed to identify whether this metric is a contributor of heterogeneity.

External validation refers to the verification of the performance of a diagnostic model, which is constructed based on the data from one hospital, with data from other hospitals, or collection of new data for model validation. Internal validation refers to the verification of model performance using data from the same hospital as that used for model construction. However, these data are often randomly shuffled such that part of the data is used for model construction and the other for model validation. Since the samples for external validation are derived from other medical institutions or newly collected samples, while those for internal validation is often randomly shuffled and selected, a subgroup analysis was conducted to determine if the difference in validation data selection was a source of heterogeneity.

Moreover, some of the original studies utilized radiomics combined with clinical risk factors to predict Ki-67 or CK-19, while others employed radiomics alone to make the predictions. Therefore, subgroup analysis was performed to assess whether these two different approaches contribute to the heterogeneity across studies.

## 3 Results

### 3.1 Study selection

**Figure 1:**
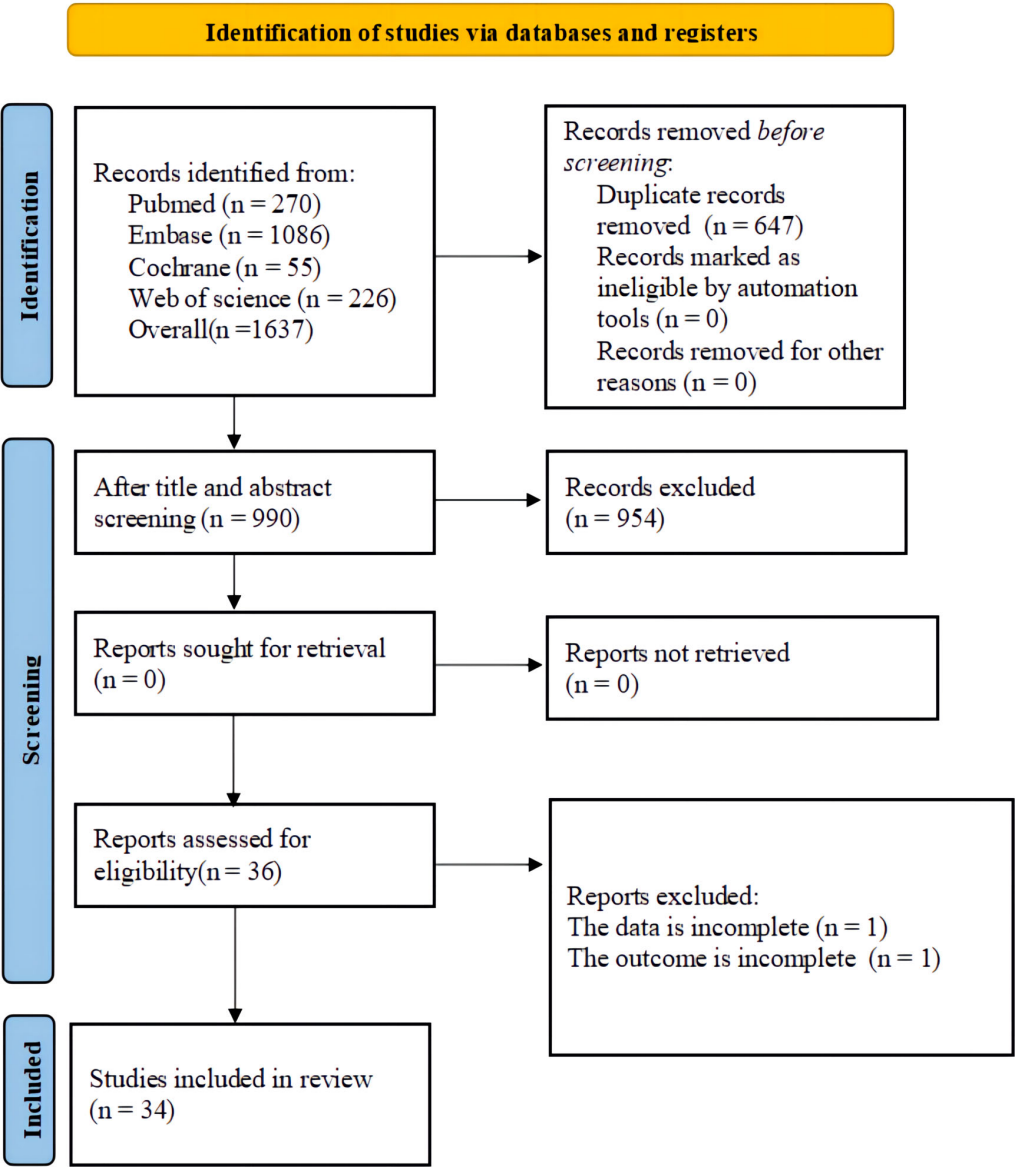
Literature screening process.

Of the initially identified 1637 studies, 647 were removed due to duplication, and 954 were excluded due to ineligibility. The remaining 36 full-text studies were independently assessed by two reviewers, and a final total of 34 studies (20–53) were included in our systematic review. The study selection process is outlined in **Figure 1**.

### 3.2 Study characteristics

A total of 4300 samples were involved (2002 samples in Ki-67 models, 2298 samples in CK-19 models) in the 34 included studies. Among the included studies, 18 (20–37) focused on Ki-67 and 16 (38–53) focused on CK-19. The samples of the included studies came from different hospitals, with six studies from multiple centers and the rest from single centers (33, 37, 42, 44, 48, 53). In all included studies, diagnostic models for Ki-67 or CK-19 were constructed based on radiomics for HCC, thus meeting the needs of this study. Positive Ki-67 expression was defined by different thresholds in different studies, with 10% label index (LI) being the main positive threshold, and the proportion of positive samples ranged from 0.30 to 0.83. The positive threshold for CK-19 was 5% LI, and the proportion of positive samples ranged from 0.14 to 0.41. Most studies were conducted in China (32 out of 34), and nearly half of them were published in 2022 and 2023 (16 out of 34). MRI was the most common radiomics source (25 out of 34), and logistic regression was the most common algorithm for model construction (28 out of 34). Nineteen studies used both radiomics and clinical risk factors for modeling whereas 15 studies used only radiomics. The basic characteristics of the included studies are provided in **Table 1**.

**Table 1:** Study characteristics.

Study	Year	Country	Output	Threshold	Sample size	Radiomics source	Feature source	Model
Xin-Xing Hu (20)	2017	China	Ki-67	10	57	MRI	R	LR
Zhao Yao (21)	2018	China	Ki-67	25	44	Ultrasound	R	SVM
Zheng Ye (22)	2019	China	Ki-67	15	89	MRI	R&C	LR
Yidi Chen (23)	2019	China	Ki-67	50	180	MRI	R	LR
Mengjie Hu (24)	2020	China	Ki-67	10	85	MRI	R&C	LR
Gaofeng Shi (25)	2020	China	Ki-67	10	52	MRI	R	LR
Hongzhen Wu (26)	2020	China	Ki-67	10	74	CT	R	LR
Zheng Ye (27)	2020	China	Ki-67	10	103	MRI	R	LR
Yanfen Fan (28)	2021	China	Ki-67	14	151	MRI	R&C	LR
Mengyuan Jing (29)	2021	China	Ki-67	10	81	MRI	R	LR
Yi Dong (30)	2022	China	Ki-67	20	101	Ultrasound	R	LR
Xumei Hu (31)	2022	China	Ki-67	20	151	MRI	R&C	SVM
Zhe Huang (32)	2022	China	Ki-67	10	120	Ultrasound	R	LR
Ziwei Liu (33)	2022	China	Ki-67	25	148	MRI	R&C	LR
Xuedong Wang (34)	2022	China	Ki-67	10	40	MRI	R	LR
Cuiyun Wu (35)	2022	China	Ki-67	20	172	CT	R&C	LR
Chuan Yan (36)	2023	China	Ki-67	10	110	MRI	R&C	LR
Linlin Zhang (37)	2023	China	Ki-67	10	244	Ultrasound	R&C	SVM
Takayuki Kawai (38)	2017	Japan	CK-19	5	98	PET/MRI	R	LR
Seo-Youn Choi (39)	2018	Korea	CK-19	5	242	MRI	R	LR
He-qing Wang (40)	2019	China	CK-19	5	78	MRI	R&C	LR
Wentao Wang (41)	2020	China	CK-19	5	227	MRI	R&C	LR
Yuying Chen (42)	2021	China	CK-19	5	141	MRI	R&C	GDBT
Fan Yang (44)	2021	China	CK-19	5	257	MRI	R&C	ANN
Zhijun Geng (43)	2021	China	CK-19	5	53	MRI	R	LR
Zheng Wanjing (45)	2020	China	CK-19	N/A	153	MRI	R&C	LR
Gao Zhiling (46)	2022	China	CK-19	5	220	MRI	R&C	LR
Yixian Guo (47)	2022	China	CK-19	5	61	MRI	R	LR
Linlin Zhang (48)	2022	China	CK-19	5	214	Ultrasound	R&C	XGBoost
Jiejun Chen (49)	2023	China	CK-19	5	73	MRI	R&C	LR
Xiaojun Hu (50)	2023	China	CK-19	5	110	MRI	R&C	LR
Mengtian Lu (51)	2023	China	CK-19	5	147	MRI	R&C	LR
Jing Lv (52)	2023	China	CK-19	N/A	66	PET/MRI	R	LR
Yue Zhao (53)	2023	China	CK-19	5	158	MRI	R&C	LR

R, radiomics; R&C, radiomics and clinical risk factors; LR, logistic regression; SVM, support vector machine; XGBoost, eXtreme Gradient Boosting; GDBT, gradient boosting decision tree; ANN, artificial neural network.

### 3.3 Quality assessment

As show in **Table 2**. All studies but one scored below 50% (18 scores) of total RQS score. Strikingly, all studies failed to register in a trial database, and none of the included studies investigated the cost-effectiveness or opened data. RQS has no qualitative quality criteria. The higher the score, the higher the quality. The scores of all studies ranged from 5 to 18, with an average of 11.91, which exceeded 30% of the total RQS score.

**Table 2:** Radiomics quality score.

Study	v1	v2	v3	v4	v5	v6	v7	v8	v9	v10	v11	v12	v13	v14	v15	v16	Score
Xin-Xing Hu (20)	1	1	0	0	-3	1	1	1	1	0	0	0	2	2	0	0	7
Zhao Yao (21)	1	1	0	0	3	1	1	0	1	0	0	2	2	2	0	0	14
Zheng Ye (22)	1	1	0	0	3	0	1	1	1	1	0	0	2	2	0	0	13
Yidi Chen (23)	1	1	0	0	-3	1	0	1	1	0	0	0	2	2	0	0	6
Mengjie Hu (24)	1	1	0	0	-3	0	0	1	1	0	0	0	2	2	0	0	5
Gaofeng Shi (25)	1	0	0	0	3	0	1	1	1	0	0	0	2	2	0	0	11
Hongzhen Wu (26)	1	0	0	0	3	0	1	1	1	0	0	0	2	2	0	0	11
Zheng Ye (27)	1	1	0	0	-3	1	0	1	1	1	0	0	2	2	0	0	7
Yanfen Fan (28)	1	1	0	0	3	1	1	1	1	1	0	2	2	2	0	0	16
Mengyuan Jing (29)	1	1	0	0	3	0	1	1	1	0	0	0	2	2	0	0	12
Yi Dong (30)	1	1	0	0	3	0	1	1	1	1	0	2	2	2	0	0	15
Xumei Hu (31)	1	0	0	0	3	1	1	0	1	0	0	2	2	2	0	0	13
Zhe Huang (32)	1	1	0	0	3	0	1	1	1	0	0	0	2	2	0	0	12
Ziwei Liu (33)	1	1	0	0	3	1	1	1	1	1	0	4	2	2	0	0	18
Xuedong Wang (34)	1	1	0	1	-3	0	0	1	1	0	0	0	2	2	0	0	6
Cuiyun Wu (35)	1	1	0	0	3	1	1	1	1	1	0	2	2	2	0	0	16
Chuan Yan (36)	1	1	0	0	3	1	1	1	1	1	0	2	2	2	0	0	16
Linlin Zhang (37)	1	1	0	0	3	1	1	0	1	0	0	4	2	2	0	0	16
Takayuki Kawai (38)	1	1	0	0	3	0	1	1	1	0	0	0	2	2	0	0	12
Seo-Youn Choi (39)	1	1	0	0	3	0	1	1	1	1	0	0	2	2	0	0	13
He-qing Wang (40)	1	1	0	0	3	1	1	0	1	0	0	0	2	2	0	0	12
Wentao Wang (41)	1	1	0	0	3	1	1	1	1	1	0	2	2	2	0	0	16
Yuying Chen (42)	1	1	0	0	3	1	1	0	1	0	0	4	2	2	0	0	16
Zhijun Geng (43)	1	1	0	0	3	0	0	1	1	0	0	0	2	2	0	0	11
Fan Yang (44)	1	1	0	0	3	1	1	0	1	0	0	4	2	2	0	0	16
Zheng Wanjing (45)	1	1	0	0	-3	0	0	1	1	0	0	0	2	2	0	0	5
Gao Zhiling (46)	1	1	0	0	-3	0	0	1	1	0	0	0	2	2	0	0	5
Yixian Guo (47)	1	1	0	0	-3	0	0	1	1	0	0	0	2	2	0	0	5
Linlin Zhang (48)	1	1	0	0	3	1	1	1	1	0	0	4	2	2	0	0	17
Jiejun Chen (49)	1	1	0	0	3	1	1	1	1	0	0	0	2	2	0	0	13
Xiaojun Hu (50)	1	1	0	0	3	1	1	1	1	0	0	0	2	2	0	0	13
Mengtian Lu (51)	1	1	0	0	3	1	1	1	1	0	0	0	2	2	0	0	13
Jing Lv (52)	1	0	0	0	3	0	0	1	1	0	0	0	2	2	0	0	10
Yue Zhao (53)	1	1	0	0	3	1	1	1	1	1	0	0	2	2	0	0	14

v1, Image protocol quality; v2, Multiple segmentations; v3, Phantom study on all scanners; v4, Imaging at multiple time points; v5, Feature reduction or adjustment for multiple testing; in the radiomics quality score (RQS); if the feature reduction or adjustment for multiple testing is not performed; 3 scores need be deducted. v6, Multivariable analysis with non-radiomics features; v7, Detect and discuss biological correlates; v8, Cut-off analyses; v9, Discrimination statistics; v10, Calibration statistics; v11, Prospective study registered in a trial database; v12, Validation; v13, Comparison to gold standard; v14, Potential clinical utility; v15, Cost-effectiveness analysis; v16, Open science and data.

### 3.4 Meta-analyses

#### 3.4.1 Ki-67

(1) *Synthesized results*

**Figure 2:**
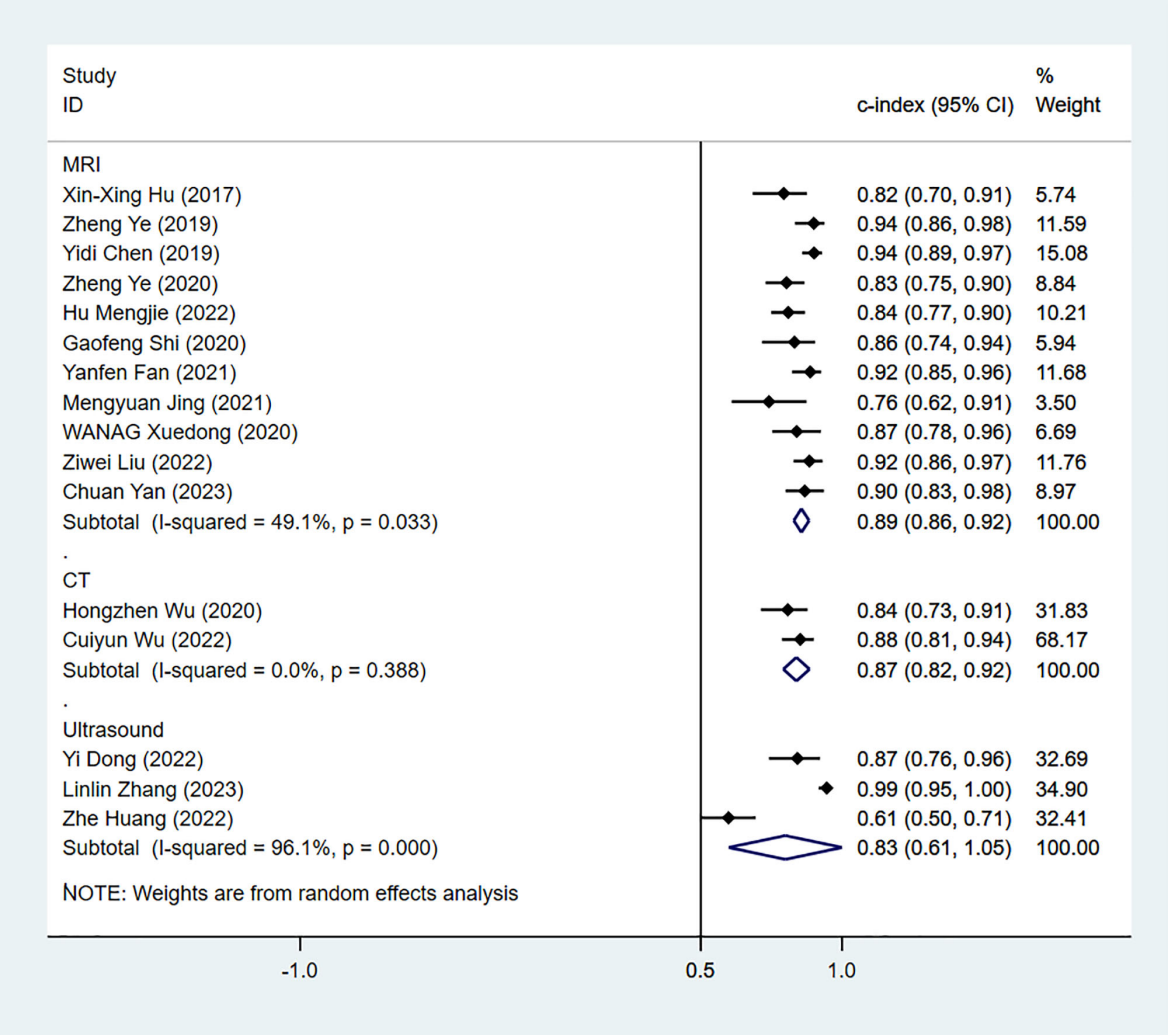
Forest plots for C-index of Ki-67 in the training set. [(1): Since the C-index estimates are synthesized, the invalid line is 0.5. (2) 95% confidence interval (CI) is depicted as the horizontal line. The hollow diamond represents the pooled C-index.].

**Figure 3:**
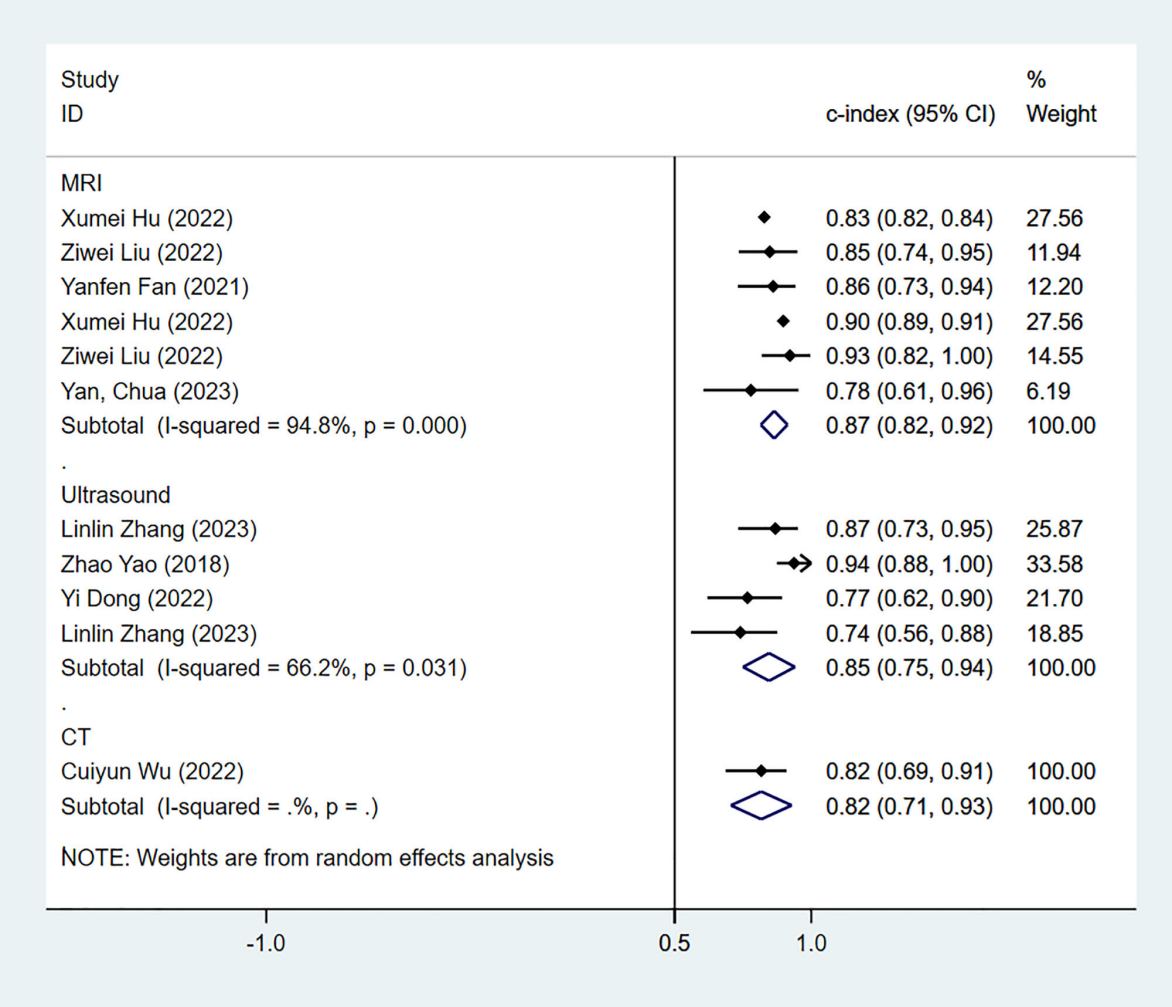
Forest plots for C-index of Ki-67 in the validation set. [(1) Since the C-index estimates are synthesized, the invalid line is 0.5. (2) 95% confidence interval (CI) is depicted as the horizontal line. The hollow diamond represents the pooled C-index.].

The results of MRI-, CT- and US-based models for predicting Ki-67 expression were synthesized separately in both the training sets (**Figure 2**) and validation sets (**Figure 3**). In the training sets, the C-index was 0.89 (95% CI:0.86–0.92) for MRI-based models, 0.87 (95% CI: 0.82–0.92) for CT-based models, and 0.83 (95% CI: 0.61–1.00) for US-based models. In the validation sets, the C-index was 0.87 (95% CI: 0.82–0.92) for MRI-based models, 0.82 (95% CI: 0.71–0.93) for CT-based models, and 0.85 (95% CI: 0.75–0.94) for US-based models.

**Figure 4:**
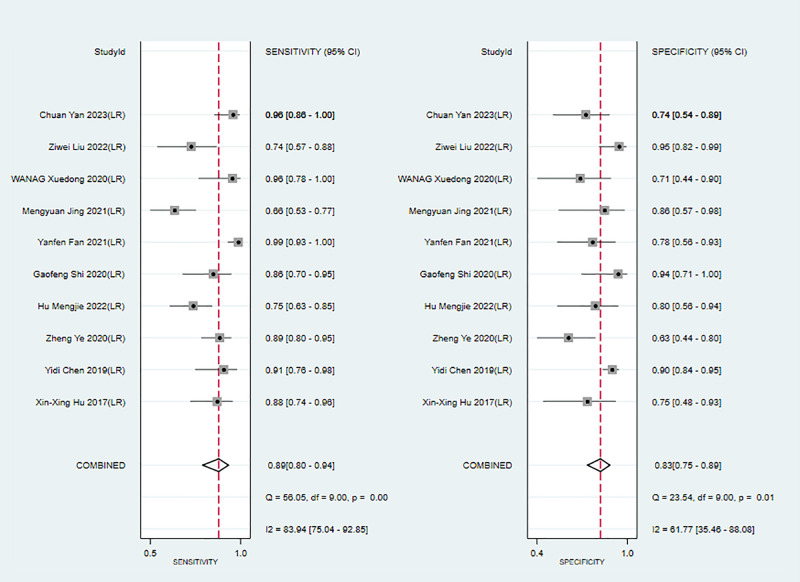
Forest plots for sensitivity and specificity of MRI-based models in the training set. [(1) Forest plots for sensitivity are shown on the left and for specificity on the right. (2) 95% confidence interval (CI) is depicted as the horizontal line. (3) The hollow diamond represents the pooled estimates.].

**Figure 5:**
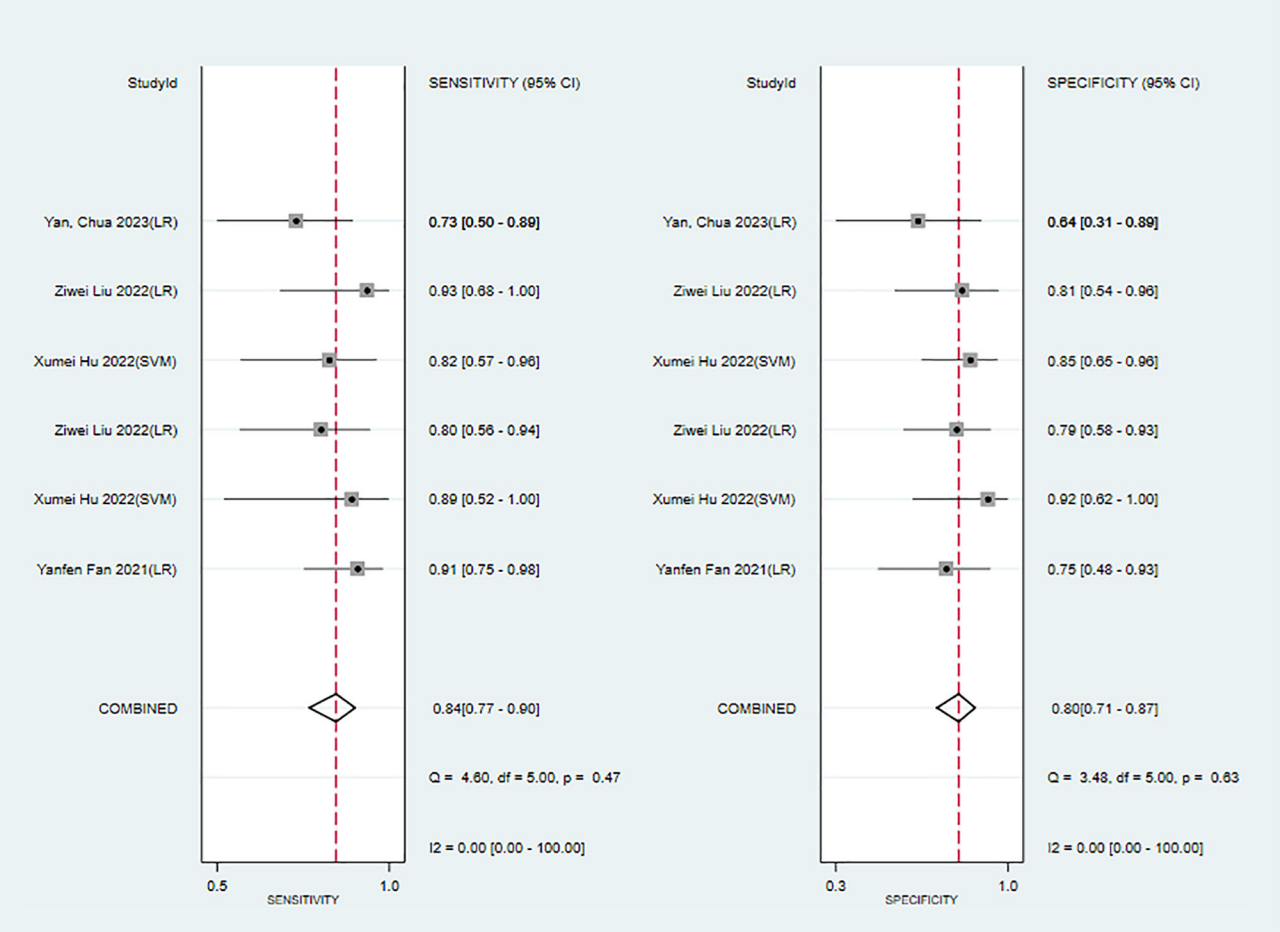
Forest plots for sensitivity and specificity of MRI-based models in the validation set. [(1) Forest plots for sensitivity are shown on the left and for specificity on the right. (2) 95% confidence interval (CI) is depicted as the horizontal line. (3) The hollow diamond represents the pooled estimates.].

In the training sets, the sensitivity and specificity of MRI-based models were 0.89 (95% CI: 0.80–0.94) and 0.83 (95% CI: 0.75–0.89), respectively (**Figure 4**). In validation sets, the sensitivity and specificity of MRI-based models were 0.84 (95% CI: 0.77–0.90) and 0.80 (95% CI: 0.71–0.81), respectively (**Figure 5**).

(2) *Subgroup analysis*

Next, we performed subgroup analyses by the positive threshold and feature category of the radiomics models in the training sets. Since CT-based models were examined in only two studies, subgroup analysis was not performed for this model type. In addition, given that the US-based models exceeded the invalid line and the synthesized results were not statistically significant, a US-based subgroup analysis was also not performed in the training sets. As shown in **Table 3**, we divided the studies based on the MRI positive Ki-67 threshold into the 10 and 10+(over 10) subgroups. The C-index of these two subgroups were 0.85 (95% CI: 0.82–0.88%) and 0.93 (95% CI: 0.91–0.96%), respectively. There was significant heterogeneity in the positive Ki-67 threshold, and C-index was increased as the threshold increased. Furthermore, subgroup analyses based on feature source, positive threshold, and validation source indicated that validation source was a significant source of heterogeneity in the MRI-based models in the validation sets (**Table 3**).

**Table 3:** Subgroup analyses in the training sets and validation sets.

Subgroups	Category	No. of studies	C-index (95% CI)		*I* ^2^(%)
Training sets of MRI					
Positive Ki-67 threshold*	10	7	0.85 (0.82–0.88)		0.0 (*P*=0.654)
	10+	4	0.93 (0.91–0.96)		0.0 (*P*=0.941)
Feature source	Radiomics and clinical	4	0.90 (0.86–0.94)		46.8 (*P*=0.131)
	Radiomics	7	0.87 (0.83–0.92)		57.8 (*P*=0.030)
Validation sets of MRI					
Validation source*	External validation	2	0.83 (0.82–0.84)		0.0 (*P*=0.718)
	Internal validation	4	0.90 (0.89–0.91)		0.0 (*P*=0.468)
Positive Ki-67 threshold	10	1	0.78 (0.61–0.96)		—
	10+	5	0.87 (0.82–0.92)		95.8 (*P*=0.000)
Feature source	Radiomics and clinical	4	0.86 (0.80–0.92)		96.8 (*P*=0.000)
	Radiomics	2	0.90 (0.89–0.91)		9.0 (*P*=0.295)
Validation sets of US					
Validation source	External validation	1	0.74 (0.58–0.90)		—
	Internal validation	2	0.87(0.78–0.97)		62.6 (*P*=0.069)
Positive Ki-67 threshold	10	2	0.82 (0.70–0.94)		41.5 (*P*=0.191)
	10+	2	0.87 (0.70–0.100)		79.9 (*P*=0.026)
Feature source	Radiomics and clinical	2	0.82 (0.70–0.94)		41.5 (*P*=0.191)
	Radiomics	2	0.87 (0.70–0.100)		66.2 (*P*=0.031)

* indicates a significant source of heterogeneity.

(3) *Reporting bias*

**Figure 6:**
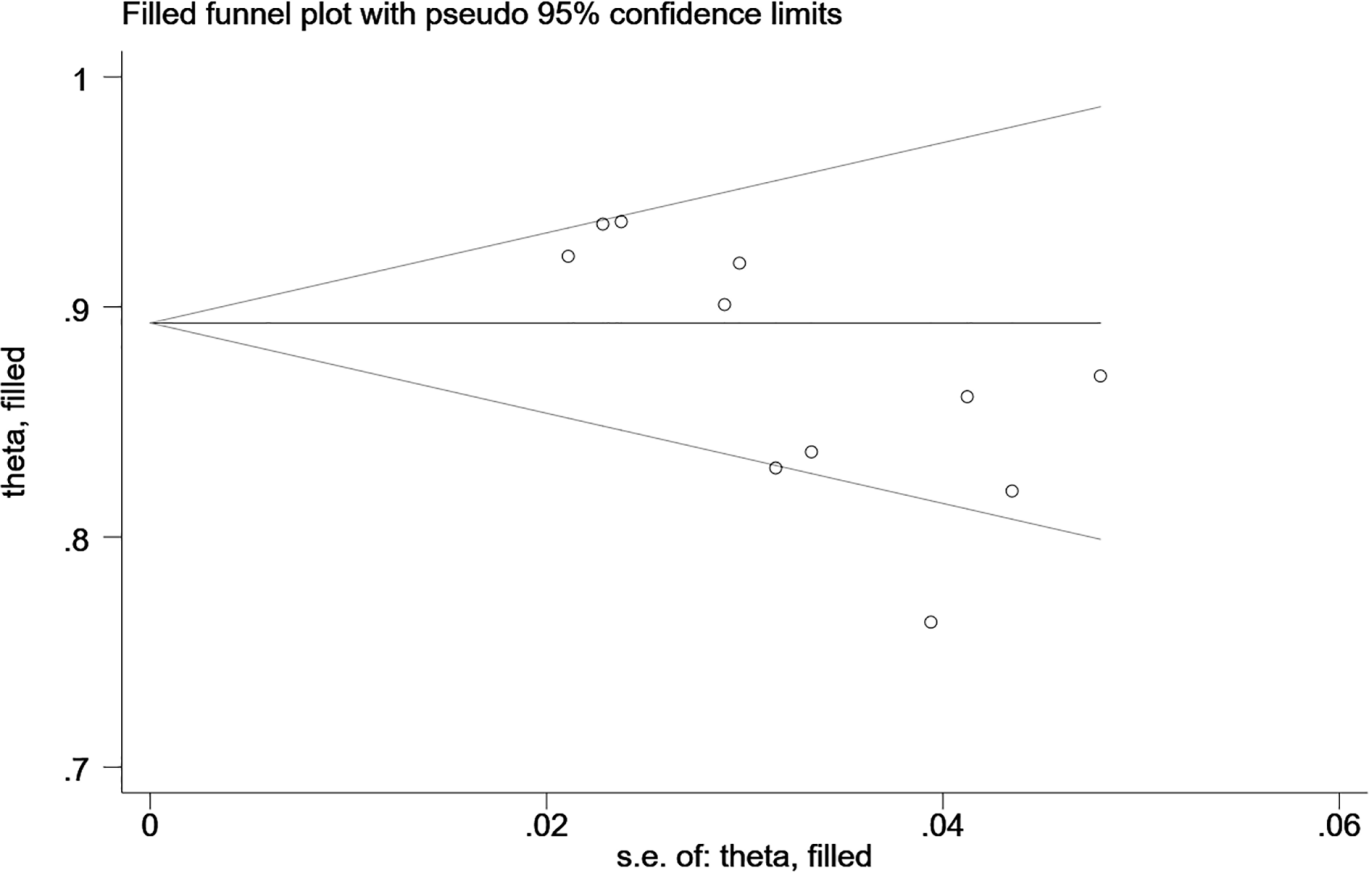
Beggs’ funnel plot of MRI-based models for predicting Ki-67 shows there is no publication bias in the MRI-based models in the training set. [(1) The ordinate represents the C-index value. (2) The abscissa represents the standard error of C-index.].

The Begg’s test showed that there was no publication bias in the MRI-based models in the training sets (*P*=0.062, continuity corrected). In addition, the funnel plot also revealed stable data without trimming (**Figure 6**).

(4) *Meta-regression*

**Figure 7:**
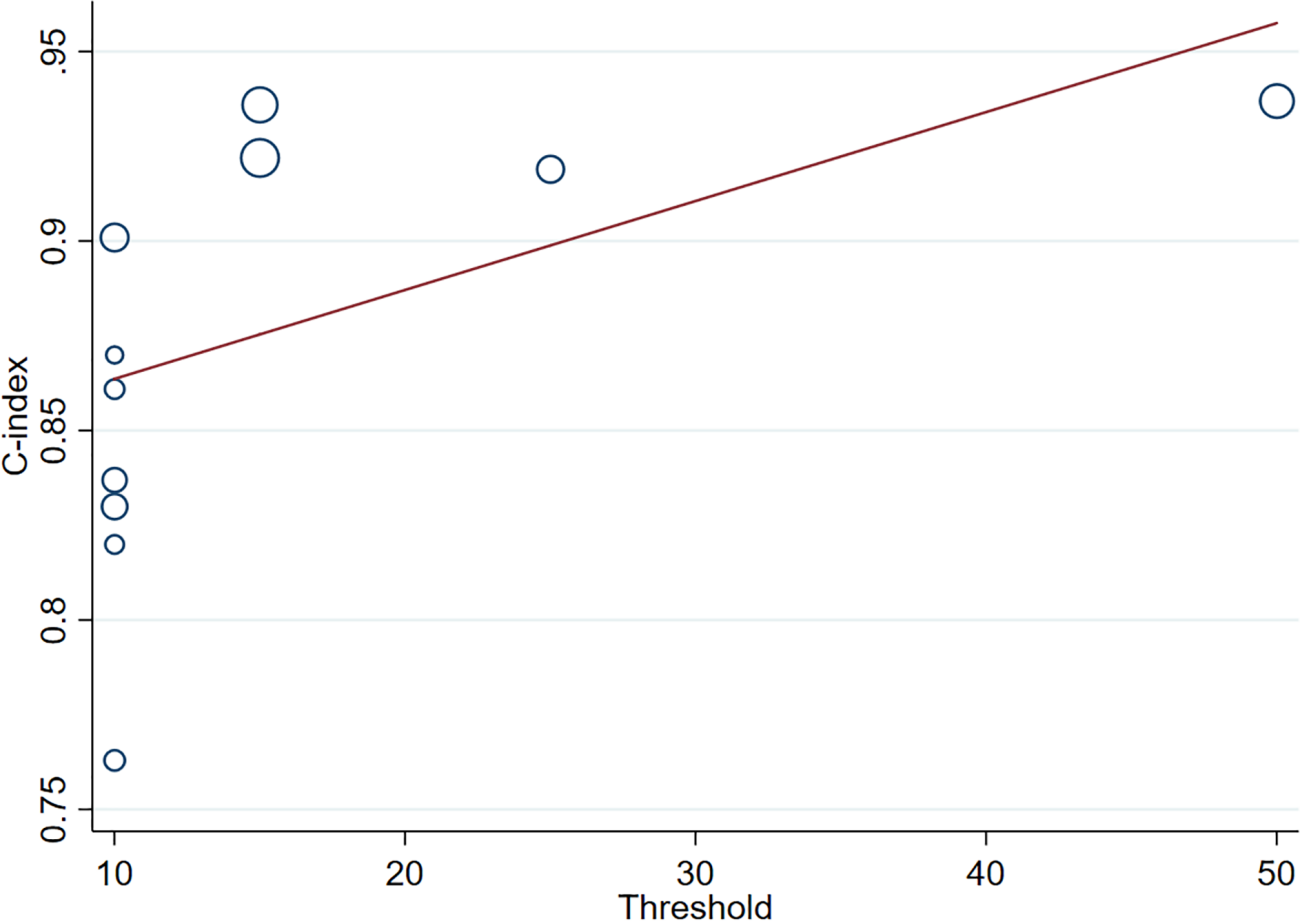
Meta-regression of Ki-67 in different positive thresholds. The abscissa represents the positive threshold of Ki-67; circles indicate the included studies, and larger circles indicate larger weights.).

We performed a meta-regression of the MRI data to further analyze the sources of heterogeneity. Our results demonstrated that the positive Ki-67 threshold was a potential source of heterogeneity since its *P*-value (*P*=0.086) was close to the significance level (0.05), which was corroborated by the results of subgroup analysis. Furthermore, meta-regression revealed that the C-index increased as Ki-67 LI increased (**Figure 7**).

#### 3.4.2 CK-19

(1) *Synthesized results*

**Figure 8:**
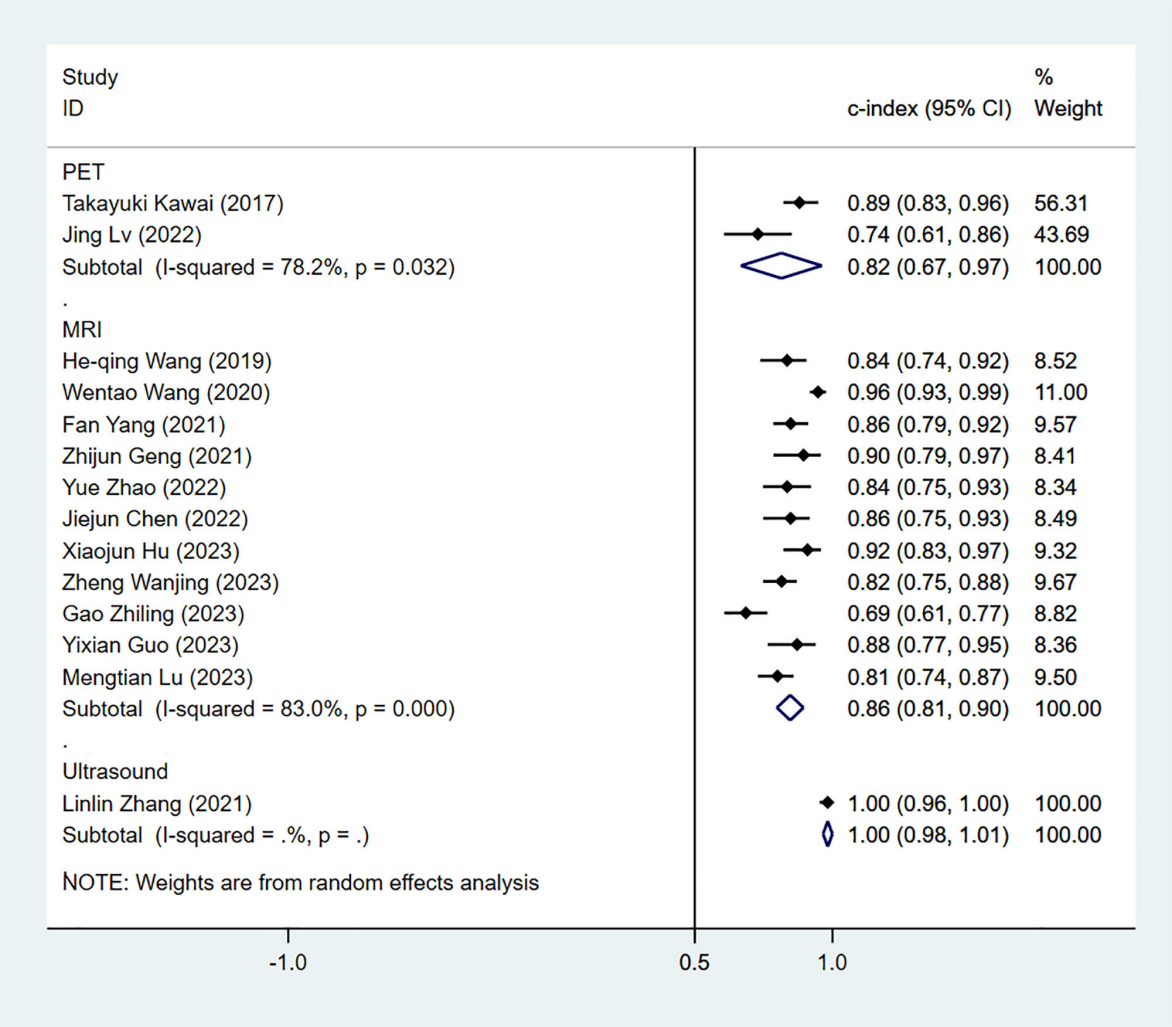
Forest plots for C-index of CK-19 in the training set. [(1) Since the C-index estimates are synthesized, the invalid line is 0.5. (2) 95% confidence interval (CI) is depicted as the horizontal line. The hollow diamond represents the pooled C-index.].

**Figure 9:**
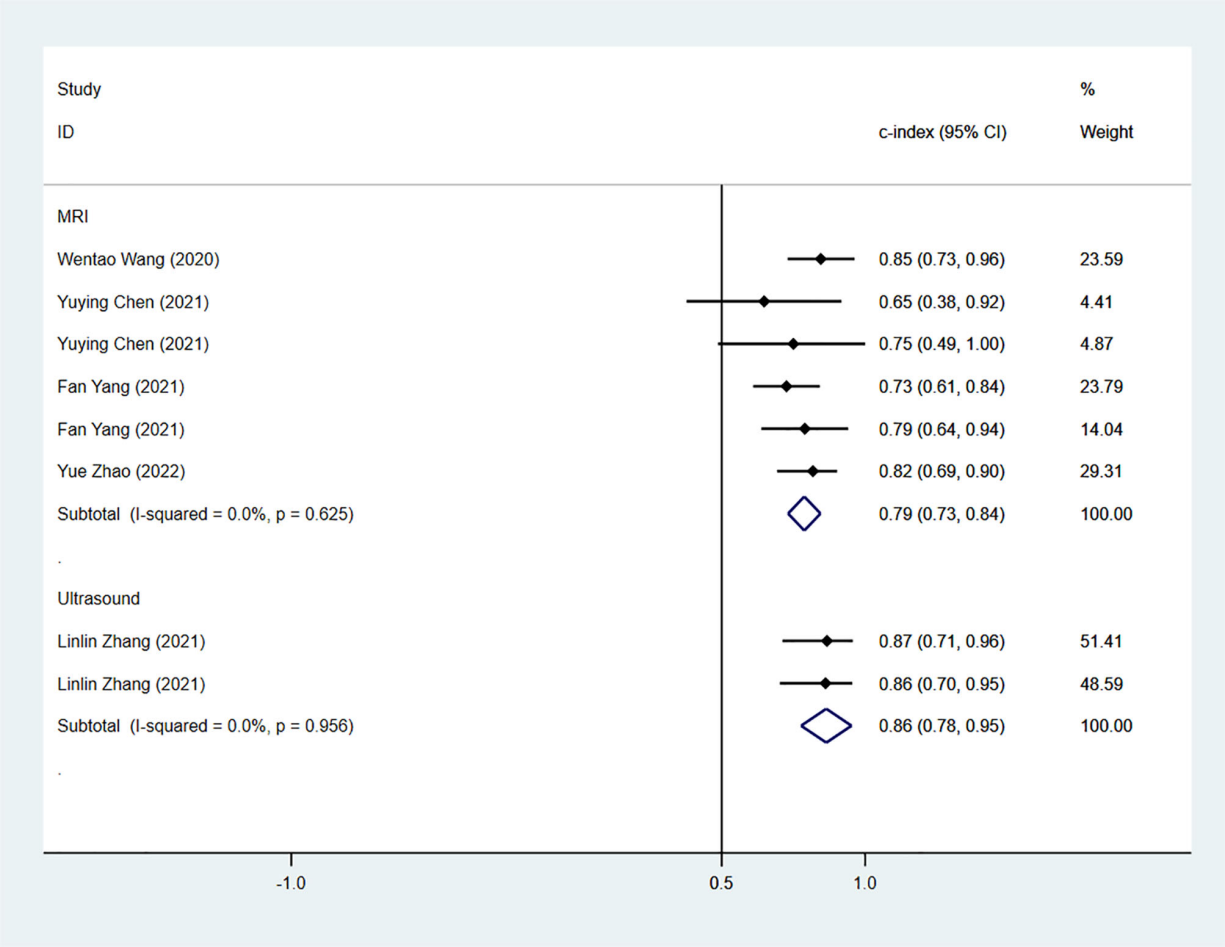
Forest plots for C-index of CK-19 in the validation set. [(1) Since the C-index estimates are synthesized, the invalid line is 0.5. (2) 95% confidence interval (CI) is depicted as the horizontal line. The hollow diamond represents the pooled C-index.].

The results of the MRI-, PET- and US-based models for predicting CK-19 expression were synthesized separately. In the training sets, the C-index was 0.86 (95% CI:0.81–0.90) for MRI-based models, 0.82 (95% CI: 0.67–0.97) for PET-based models, and 1.00 (95% CI: 0.98–1.00) for US-based models (**Figure 8**). In the validation sets, the C-index was 0.79 (95% CI: 0.73–0.84) for the MRI-based models (**Figure 9**).

**Figure 10:**
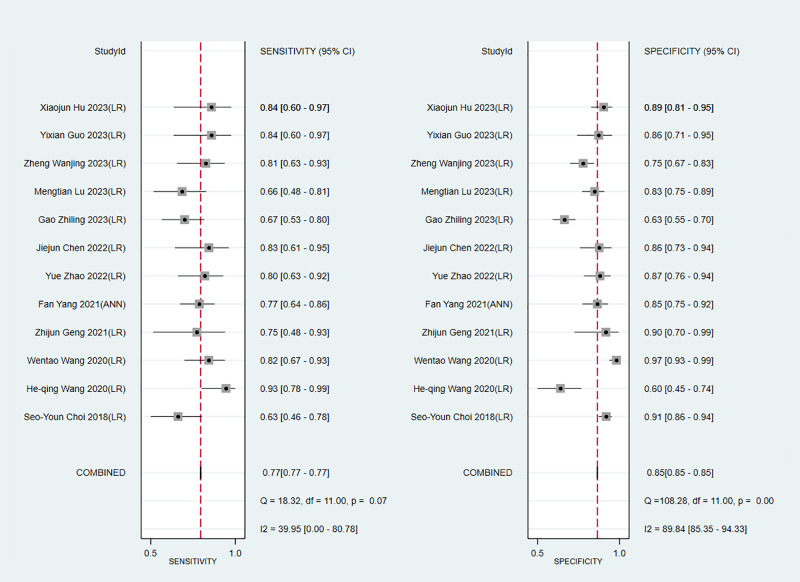
Forest plots for sensitivity and specificity of CK-19 in the training set. [(1) Forest plots for sensitivity are shown on the left and for specificity on the right. (2) 95% confidence interval (CI) is depicted as the horizontal line. (3) The hollow diamond represents the pooled estimates.].

**Figure 11:**
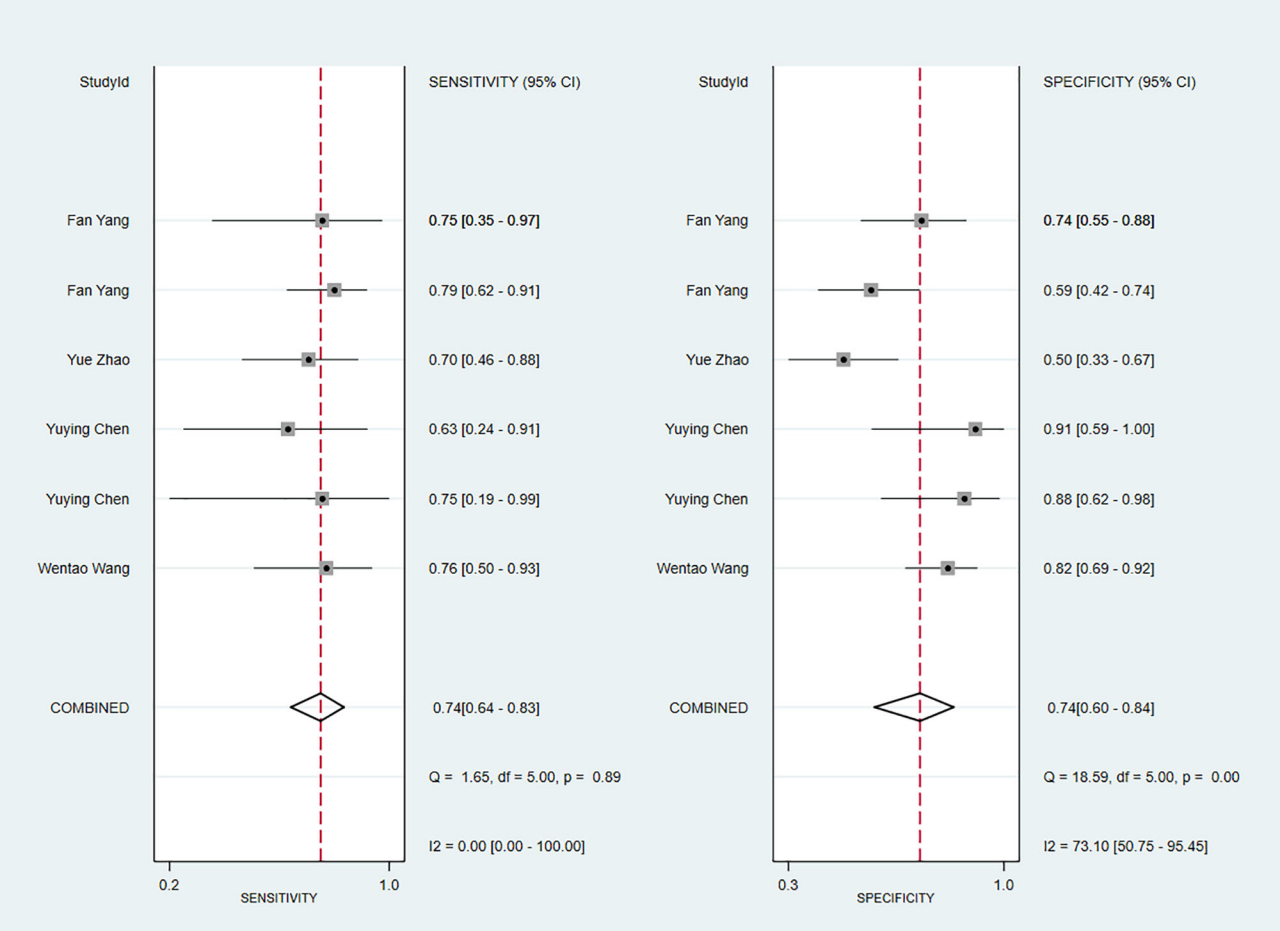
Forest plots for sensitivity and specificity of CK-19 in the validation set. [(1) Forest plots for sensitivity are shown on the left and for specificity on the right. (2) 95% confidence interval (CI) is depicted as the horizontal line. (3) The hollow diamond represents the pooled estimates.].

We next determined the diagnostic sensitivity and specificity of the MRI-based models using a bivariate mixed-effects model. Our data showed that the sensitivity and specificity of MRI-based models were 0.89 (95% CI: 0.80–0.94) and 0.83 (95% CI: 0.75–0.89) in training sets (**Figure 10**), and 0.84 (95% CI: 0.77–0.90) and 0.80 (95% CI: 0.71–0.81) in the validation sets, respectively (**Figure 11**).

(2) *Subgroup analysis and sensitivity analysis*

**Figure 12:**
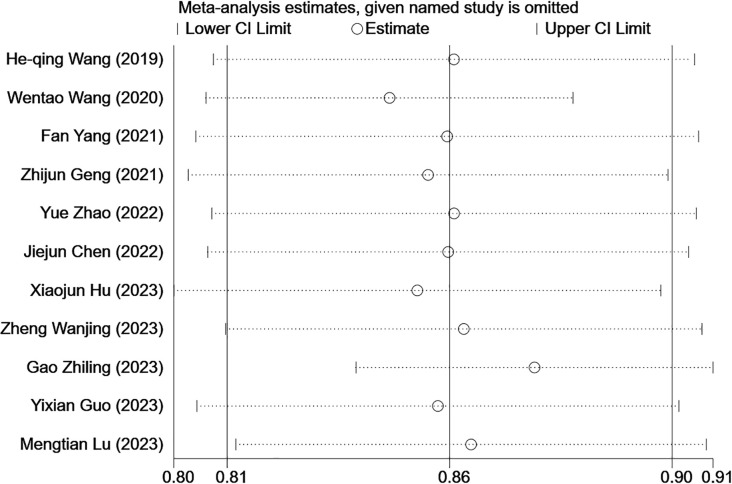
Sensitivity analysis of CK-19 in the training set confirmed that the pooled C-index results were stable. [(1) The ordinate represents the removal of one study. (2) The abscissa represents the pooled C-index after the removal of one study. (3) 95% confidence interval (CI) is depicted as the horizontal line.].

In the training sets, subgroup analysis based on feature source revealed that C-index was 0.85 (95% CI: 0.77–0.92) for models based on radiomics and clinical features and 0.86 (95% CI: 0.81–0.90) for models based on radiomics features only. The *I* ^2^ for models based on radiomics and clinical features (88.7% *P*=0.000) and those based on radiomics only (0.0% *P*=0.468) implied the feature source was not a significant source of heterogeneity. Further sensitivity analysis confirmed that the synthesized C-index results were stable (**Figure 12**).

In the validation sets, C-index was 0.75 (95% CI: 0.66–0.84) for external validation, 0.82 (95% CI: 0.74–0.89) for internal validation, 0.81 (95% CI: 0.74–0.88) for models based on radiomics and clinical features, and 0.75 (95% CI: 0.66–0.84) for models based on radiomics features (**Table 4**).

**Table 4:** Subgroup analysis of CK-19 in training sets and validation sets.

Subgroups	Category	No. of studies	C-index (95% CI)	*I* ^2^(%)
Training sets of MRI				
Feature source	Radiomics and clinical	7	0.85 (0.77–0.92)	88.7 (*P*=0.000)
	Radiomics	4	0.86 (0.81–0.90)	0.0 (*P*=0.468)
Validation sets of MRI				
Validation source	External validation	2	0.75 (0.66–0.84)	0.0 (*P*=0.417)
	Internal validation	4	0.82 (0.74–0.89)	0.0 (*P*=0.805)
Feature source	Radiomics and clinical	4	0.81 (0.74–0.88)	0.0 (*P*=0.575)
	Radiomics	2	0.75 (0.66–0.84)	0.0 (*P*=0.510)

(3) *Reporting bias*

**Figure 13:**
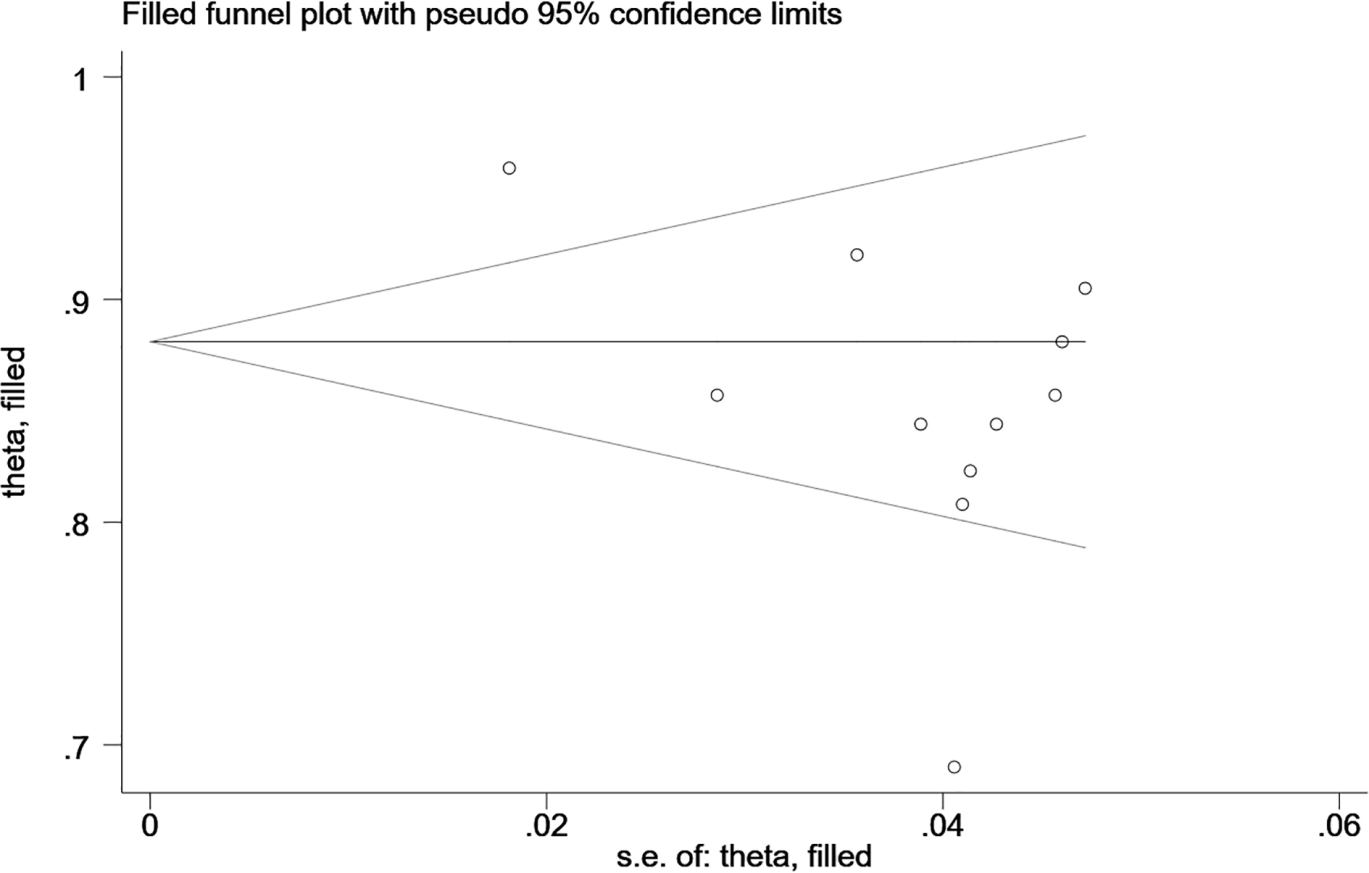
Beggs’ funnel plot of MRI-based models for predicting CK-19 shows there is no publication bias in the MRI-based models in the training sets. [(1) The ordinate represents the C-index value. (2) The abscissa represents the standard error of C-index.].

The Begg’s test showed no publication bias in the MRI-based models in the training sets (*P*=0.755, continuity corrected). In addition, the funnel plot also revealed stable data without trimming (**Figure 13**).

### 3.5 Apparent diffusion coefficient

**Figure 14:**
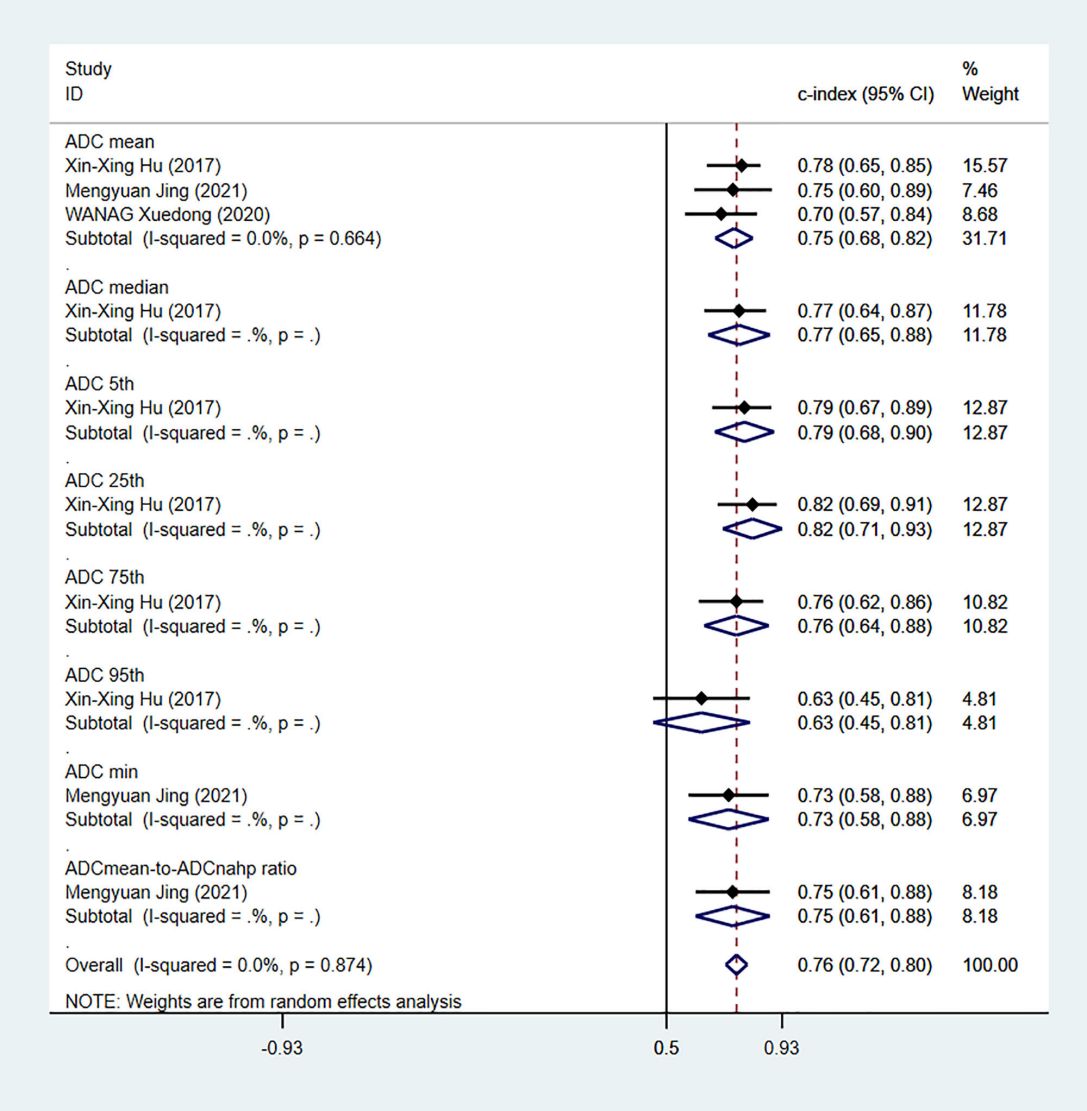
Forest plots for C-index of ADC in the training sets. [(1) Since the C-index estimates are synthesized, the invalid line is 0.5. (2) 95% confidence interval (CI) is depicted as the horizontal line. The hollow diamond represents the pooled C-index.].

**Figure 15:**
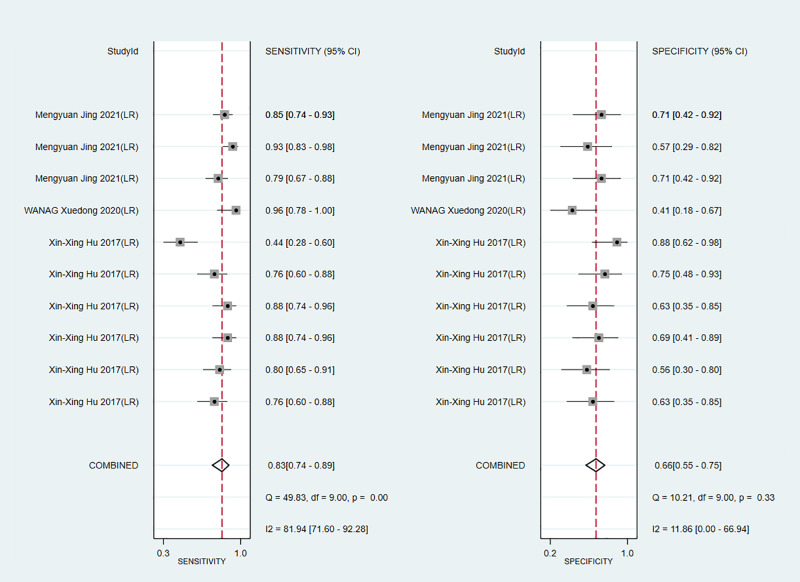
Forest plots for sensitivity and specificity of ADC in the training set. [(1) Forest plots for sensitivity are shown on the left and for specificity on the right. (2) 95% confidence interval (CI) is depicted as the horizontal line. (3) The hollow diamond represents the pooled estimates.].

Apparent diffusion coefficient (ADC) value is a highly specific diagnostic marker for Ki-67. We pooled ADC values from three studies (only in training sets) and found that C-index, sensitivity and specificity of the MRI-based models were 0.76(95% CI: 0.72–0.80), 0.83 (95% CI: 0.74–0.89) and 0.66(95% CI: 0.55–0.75), respectively (**Figures 14**, **15**).

## 4 Discussion

### 4.1 Summary of the main findings

This review shows that there is no clear consensus on the cutoff for positive Ki-67 expression, and a 10% LI score is the common threshold used for defining positive Ki-67 expression (10 out of 18). A previous systematic review has reported that a higher Ki-67 LI indicates a faster progression and poorer prognosis in HCC patients (54). HCC patients with Ki-67 LI > 25% are more susceptible to recurrence after surgery (33), and the mean Ki-67 LI of poorly differentiated HCC is higher than that of well- and moderately differentiated HCC (55). Meanwhile, our pooled results of C-index also suggested that 10% LI score also has a good diagnostic performance in radiomics models. Therefore, this review recommends a 10% LI score as a positive threshold for high Ki-67 expression in radiomics model to aid in early intervention in HCC patients.

ADC value is an important radiomics feature used for predicting MVI and tumor grade in HCC (56). It has also been reported that ADC value is negatively correlated with positive Ki-67 expression and is a predictor for Ki-67 expression in HCC (29, 57). However, compared ADC value pooled results with other pooled results in the study, the diagnostic performance of ADC value is poor. Therefore, it is not recommended to use only a single ADC value for the diagnosis of Ki-67.

Radiomics has demonstrated promising application prospects in differentiating HCC from other solid lesions, and for predicting MVI, early recurrence after hepatectomy, and prognosis after locoregional or systemic therapies (58). Our findings further confirmed that radiomics is a useful non-invasive diagnostic approach for determining Ki-67 and CK-19 expression status and a promising complementary technique to biopsy.

It is worth noting that most of the included studies were from China, which is likely attributed to the high incidence of liver cancer in China (59). Therefore, there is a more urgent need for liver cancer research in China, which may explain why these studies occurred in China.

Based on the evidence from this study, 5% LI is identified as the positive threshold of CK-19, and 10% LI can be used as the positive threshold of Ki-67 for the radiomics model and may be helpful for early intervention of HCC. In terms of radiomics source, MRI is the most extensively studied, regardless of Ki-67 or CK-19. In addition, this study also showed that models combining clinical risk factors and radiomics had higher C-index upper limit, which indicates that the incorporation of clinical risk factors can improve the accuracy of radiomics models in clinical practice.

Taken together, this study suggests that the MRI-based radiomics plus clinical risk factor model can be used for predicting Ki-67 and CK-19 expression in HCC using 10% LI and 5% LI as the positive thresholds for the two markers, respectively.

### 4.2 Comparison of MRI and US

MRI provides multi-directional, multi-sequence and high-resolution imaging with a plethora of radiomics features. However, MRI is expensive and has a prolonged examination time, which may not be suitable for some patients with metal implants. Compared with MRI, US is less costly, shorter in examination time, and not affected by metals in the patient. However, US examination is more subjective and prone to limitations in the operator’s technique and experience. Our study demonstrated that both US- and MRI-based models have favorable predictive accuracy and can hence be selected according to clinical need.

### 4.3 Comparison with previous studies

This is to date the first study that systematically evaluates the diagnostic value of radiomics in Ki-67 and CK-19 expression in HCC. A review by Chalkidou et al. indicated that Ki-67 expression was strongly correlated with many cancer types, including brain, lung and breast cancers (60). However, they did not examine the association between Ki-67 and HCC. Other related reviews suggested that high CK-19 or Ki-67 expression was significantly associated with poor prognosis in HCC patients (13, 54). These reviews mainly assessed the prognostic value but not the diagnostic value of radiomics in CK-19 and Ki-67 in HCC.

Despite a lack of comparisons between radiomics and other methods such as immunohistochemistry in current research, radiomics still is a promising non-invasive diagnostic tool for HCC and may serve as a useful complementary approach in patients with contraindications for biopsy or dynamic monitoring after treatment.

### 4.4 Strengths and limitations

This is the first meta-analysis to quantitatively assess the diagnostic performance of radiomics in predicting Ki-67 or CK-19 expression status and may provide key clues for the further clinical application of radiomics in HCC.

However, several limitations must be considered. First, we included 34 studies, but the external validation of radiomic models was only performed in few studies. Hence, more prospective multicenter trials are needed to fully validate the diagnostic value of radiomics. Second, different inclusion criteria were used in the included studies, making it difficult to determine what clinical settings these results are applicable to. Third, most of the studies included patients with different stages of HCC, which may represent a confounding factor that affects the performance of radiomics model in diagnosis. Fourth, given that a random effects model was used for meta-analysis, the results could possibly be biased with large CIs. Fifth, the threshold for positive Ki-67 expression varied across studies. Although we performed meta-analyses based on different threshold values, some thresholds of Ki-67 were reported in a small number of the studies, which may limit the interpretation of the results. Sixth, most radiomics models were based on MRI, with only a few on US and CT, which may impact the robustness of the pooled results. Therefore, a larger sample size is warranted to enhance the robustness of the results for US- and CT-based models. Last, since most studies originated from Asia, there may be geographic bias in our results.

In addition, we note that at this stage, the included studies mainly focused on the construction of diagnostic models based on radiomics, and lacked the establishment of radiomics score based on the radiomics variables. Therefore, in future studies, researchers should try to establish a radiomics score to analyze the non-linear association between variables and outcome.

## 5 Conclusions

MRI-based radiomics is a promising non-invasive diagnostic tool for predicting positive Ki-67 and CK-19 expression in HCC patients. A standard method for constructing radiomics models should be established in future studies to ensure consistency. In addition, since some of the items of the RQS were difficult to implement, further updates of the RQS may be warranted in subsequent studies. Moreover, the threshold for positive Ki-67 expression remains controversial, and thus as reasonable threshold that affects prognosis will need to be identified.

## Data availability statement

The original contributions presented in the study are included in the article/**Supplementary Material**. Further inquiries can be directed to the corresponding author.

## Author contributions

LZ: Conceptualization, Data curation, Formal Analysis, Methodology, Writing – original draft, Writing – review & editing. YC: Conceptualization, Data curation, Formal Analysis, Methodology, Writing – original draft, Writing – review & editing. YL: Resources, Software, Supervision, Writing – original draft. CW: Resources, Software, Writing – original draft. CX: Investigation, Supervision, Writing – original draft. XW: Conceptualization, Formal Analysis, Methodology, Writing – review & editing.
