# Supplementary material for: Diagnostic value of radiomics in predicting Ki-67 and cytokeratin 19 expression in hepatocellular carcinoma: a systematic review and meta-analysis
Source: Front Oncol. 2024 Jan 3;13:1323534. doi: 10.3389/fonc.2023.1323534 (PMC10792117; doi:10.3389/fonc.2023.1323534)
Supplement: Supplementary file 1 [file Table_1.docx]

# Supplementary material

**Diagnostic Value of Radiomics in Predicting Ki-67 and Cytokeratin 19 Expression in Hepatocellular Carcinoma: A Systematic Review and Meta-analysis**

**Lu Zhou MD, Yiheng Chen MD, Yan Li MD, Chaoyong Wu MM, Chongxiang Xue MM, Xihong Wang MD**

***Corresponding author**: Xihong Wang

Email: wangxihong@hactcm.edu.cn

**Table S1 Literature search strategy**

**1.Pubmed**

| Search number | Query | Results |
| --- | --- | --- |
| 1 | (Carcinoma, Hepatocellular[MeSH Terms]) OR (Hepatocellular Carcinoma*[Title/Abstract]) OR (Liver Cancer*[Title/Abstract]) OR (Liver Cell Carcinoma*[Title/Abstract]) OR (Hepatocellular Carcinoma*[Title/Abstract]) OR (Hepatoma*[Title/Abstract]) OR (hepatic carcinoma*[Title/Abstract]) OR (hepatic cell carcinoma*[Title/Abstract]) OR (hepatocarcinoma*[Title/Abstract]) OR (hepatocellular carcinoma*[Title/Abstract]) OR (hepatoma*[Title/Abstract]) OR (liver carcinoma*[Title/Abstract]) OR (liver carcinoma rupture[Title/Abstract]) OR (malignant hepatoma*[Title/Abstract]) OR (primary liver carcinoma*[Title/Abstract]) OR (HCC[Title/Abstract]) OR (HCCs[Title/Abstract]) OR (hepatic cancer*[Title/Abstract]) OR (hepatic neoplasia[Title/Abstract]) OR (hepatic neoplasm*[Title/Abstract]) OR (hepatic tumor*[Title/Abstract]) OR (hepatic tumour*[Title/Abstract]) OR (hepatocellular cancer*[Title/Abstract]) OR (hepatocellular neoplasia[Title/Abstract]) OR (hepatocellular neoplasm*[Title/Abstract]) OR (hepatocellular tumor*[Title/Abstract]) OR (hepatocellular tumour*[Title/Abstract]) OR (liver cell tumor*[Title/Abstract]) OR (liver cell tumour*[Title/Abstract]) OR (liver neoplasia[Title/Abstract]) OR (liver neoplasm*[Title/Abstract]) OR (liver tumour*[Title/Abstract]) OR (liver tumor*[Title/Abstract]) OR (hepatic malignancies[Title/Abstract]) OR (hepatic malignancy[Title/Abstract]) OR (hepatocellular malignancies[Title/Abstract]) OR (hepatocellular malignancy[Title/Abstract]) OR (liver cell cancer*[Title/Abstract]) OR (liver malignancies[Title/Abstract]) OR (liver malignancy[Title/Abstract]) OR (malignant hepatic tumor*[Title/Abstract]) OR (malignant hepatic tumour*[Title/Abstract]) OR (malignant liver neoplasm*[Title/Abstract]) OR (malignant liver tumor*[Title/Abstract]) OR (malignant liver tumour*[Title/Abstract]) OR (primary liver cancer*[Title/Abstract]) | 200,818 |
| 2 | (Tomography, X-Ray Computed[MeSH Terms]) OR (Computer Assisted Tomography[Title/Abstract]) OR (Computerized Axial Tomography[Title/Abstract]) OR (computerised axial tomography[Title/Abstract]) OR (computed axial tomography[Title/Abstract]) OR (CAT[Title/Abstract]) OR (computerised tomography[Title/Abstract]) OR (Computerized Tomography[Title/Abstract]) OR (Computed Tomography[Title/Abstract]) OR (computer tomography[Title/Abstract]) OR (computed tomographic[Title/Abstract]) OR (CT[Title/Abstract]) OR (Magnetic Resonance Imaging[MeSH Terms]) OR (Magnetic Resonance Imaging[Title/Abstract]) OR (Magnetic Resonance Image*[Title/Abstract]) OR (MRI[Title/Abstract]) OR (mr imaging[Title/Abstract]) OR (nuclear magnetic resonance imaging[Title/Abstract]) OR (NMR imaging[Title/Abstract]) OR (magnetic resonance tomography[Title/Abstract]) OR (MR Tomography[Title/Abstract]) OR (nuclear magnetic resonance Tomography[Title/Abstract]) OR (NMR Tomography[Title/Abstract]) OR (Radiomics[Title/Abstract]) OR (radiomic[Title/Abstract]) OR (radiogenomic[Title/Abstract]) OR (radiomics-based[Title/Abstract]) OR (Texture[Title/Abstract]) OR (machine learning[Title/Abstract]) OR (Transfer Learning[Title/Abstract]) OR (Deep learning[Title/Abstract]) OR (Ensemble Learning[Title/Abstract]) OR (artificial intelligence[Title/Abstract]) OR (random forest[Title/Abstract]) OR (neural network[Title/Abstract]) OR (neural networks[Title/Abstract]) OR (CNN[Title/Abstract]) OR (Support vector machine[Title/Abstract]) OR (SVM[Title/Abstract]) OR (Gradient Boosting Machine[Title/Abstract]) OR (Nomogram[Title/Abstract]) OR (XGBoost[Title/Abstract]) OR (Adaboost[Title/Abstract]) OR (Decision tree[Title/Abstract]) OR (ResNet-50[Title/Abstract]) OR (ResNet[Title/Abstract]) OR (Naive Bayesian[Title/Abstract]) OR (Multilayer perceptron[Title/Abstract]) OR (Bayesian network[Title/Abstract]) OR (Transformer[Title/Abstract]) OR (DNN[Title/Abstract]) OR (RNN[Title/Abstract]) | 1,777,782 |
| 3 | (Ki-67 Antigen[MeSH Terms]) OR (Ki 67 Antigen[Title/Abstract]) OR (Ki-67 Antigen[Title/Abstract]) OR (Ki-67[Title/Abstract]) OR (Ki67[Title/Abstract]) OR (Ki 67[Title/Abstract]) OR (MIB-1[Title/Abstract]) OR (MIB1[Title/Abstract]) OR (MIB 1[Title/Abstract]) OR (Antigen Ki-67[Title/Abstract]) OR (Antigen Ki 67[Title/Abstract]) OR (MIB-1 Antigen[Title/Abstract]) OR (MIB 1 Antigen[Title/Abstract]) OR (Antigen Ki67[Title/Abstract]) | 46,487 |
| 4 | (Keratin-19[MeSH Terms]) OR (Keratin 19[Title/Abstract]) OR (Keratin-19[Title/Abstract]) OR (Cytokeratin-19[Title/Abstract]) OR (Cytokeratin 19[Title/Abstract]) OR (Ck-19[Title/Abstract]) OR (Ck19[Title/Abstract]) OR (Ck 19[Title/Abstract]) | 5,713 |
| 5 | #1 AND #2 AND #3 | 114 |
| 6 | #1 AND #2 AND #4 | 89 |
| 7 | ((Ultrasonic[MeSH Terms]) OR (Ultrasonography[MeSH Terms]) OR (Ultrasound*[Title/Abstract]) OR (Echotomography[Title/Abstract]) OR (Ultrasonic[Title/Abstract]) OR (Sonography[Title/Abstract]) OR (Ultrasonographic[Title/Abstract]) OR (Echography[Title/Abstract]) OR (Ultrasonography[Title/Abstract]) OR (phonophoresis[Title/Abstract]) OR (sonication[Title/Abstract]) OR (sonification[Title/Abstract]) OR (ultra sound[Title/Abstract]) OR (doptone[Title/Abstract]) OR (echography[Title/Abstract]) OR (echogram[Title/Abstract]) OR (echographic[Title/Abstract]) OR (echoscopy[Title/Abstract]) OR (echosound[Title/Abstract]) OR (sonogram[Title/Abstract]) OR (sonographic[Title/Abstract]) OR (sonographic[Title/Abstract]) OR (sonography[Title/Abstract]) OR (ultrasonogram[Title/Abstract]) OR (ultrasonographic[Title/Abstract]) OR (ultrasonography[Title/Abstract]) OR (echography[Title/Abstract]))AND ("1000/01/01"[Date - Entry] : "2023/05/10"[Date - Entry]) | 745,489 |
| 8 | #1 AND #3 AND #7 | 42 |
| 9 | #1 AND #4 AND #7 | 25 |

**2.Cochrane**

| Search number | Query | Results |
| --- | --- | --- |
| #1 | MeSH descriptor: [Carcinoma, Hepatocellular] explode all trees | 2384 |
| #2 | ('Hepatocellular Carcinoma*' OR 'Liver Cancer*' OR 'Liver Cell Carcinoma*' OR 'Hepatocellular Carcinoma*' OR 'Hepatoma*' OR 'hepatic carcinoma*' OR 'hepatic cell carcinoma*' OR 'hepatocarcinoma*' OR 'hepatocellular carcinoma*' OR 'hepatoma*' OR 'liver carcinoma*' OR 'liver carcinoma rupture' OR 'malignant hepatoma*' OR 'primary liver carcinoma*' OR 'HCC' OR 'HCCs' OR 'hepatic cancer*' OR 'hepatic neoplasia' OR 'hepatic neoplasm*' OR 'hepatic tumor*' OR 'hepatic tumour*' OR 'hepatocellular cancer*' OR 'hepatocellular neoplasia' OR 'hepatocellular neoplasm*' OR 'hepatocellular tumor*' OR 'hepatocellular tumour*' OR 'liver cell tumor*' OR 'liver cell tumour*' OR 'liver neoplasia' OR 'liver neoplasm*' OR 'liver tumour*' OR 'liver tumor*' OR 'hepatic malignancies' OR 'hepatic malignancy' OR 'hepatocellular malignancies' OR 'hepatocellular malignancy' OR 'liver cell cancer*' OR 'liver malignancies' OR 'liver malignancy' OR 'malignant hepatic tumor*' OR 'malignant hepatic tumour*' OR 'malignant liver neoplasm*' OR 'malignant liver tumor*' OR 'malignant liver tumour*' OR 'primary liver cancer*'):ti,ab,kw | 22472 |
| #3 | MeSH descriptor: [Magnetic Resonance Imaging] explode all trees | 10854 |
| #4 | MeSH descriptor: [Tomography, X-Ray Computed] explode all trees | 7244 |
| #5 | ('Computer Assisted Tomography' OR 'Computerized Axial Tomography' OR 'computerised axial tomography' OR 'computed axial tomography' OR 'CAT' OR 'computerised tomography' OR 'Computerized Tomography' OR 'Computed Tomography' OR 'computer tomography' OR 'computed tomographic' OR 'CT' OR 'nuclear magnetic resonance imaging' OR 'Magnetic Resonance Imaging' OR 'Magnetic Resonance Image*' OR 'MRI' OR 'mr imaging' OR 'NMR imaging' OR 'magnetic resonance tomography' OR 'MR Tomography' OR 'nuclear magnetic resonance Tomography' OR 'NMR Tomography' OR 'radiomic*' OR 'radiogenomic' OR 'radiomics-based' OR 'Texture' OR 'machine learning' OR 'Transfer Learning' OR 'Deep learning' OR 'Ensemble Learning' OR 'artificial intelligence' OR 'random forest' OR 'neural network' OR 'neural networks' OR 'CNN' OR 'Support vector machine' OR 'SVM' OR 'Gradient Boosting Machine' OR 'Nomogram' OR 'XGBoost' OR 'Adaboost' OR 'Decision tree' OR 'ResNet-50' OR 'ResNet' OR 'Naive Bayesian' OR 'Multilayer perceptron' OR 'Bayesian network' OR 'Transformer' OR 'DNN' OR 'RNN'):ti,ab,kw | 145812 |
| #6 | MeSH descriptor: [Ki-67 Antigen] explode all trees | 332 |
| #7 | ('Ki 67 Antigen' OR 'Ki-67 Antigen' OR 'Ki-67' OR 'Ki67' OR 'Ki 67' OR 'MIB-1' OR 'MIB1' OR 'MIB 1' OR 'Antigen Ki-67' OR 'Antigen Ki 67' OR 'MIB-1 Antigen' OR 'MIB 1 Antigen' OR 'Antigen Ki67'):ti,ab,kw | 2281 |
| #8 | MeSH descriptor: [Keratin-19] explode all trees | 41 |
| #9 | ('Keratin 19' OR 'Keratin-19' OR 'Cytokeratin-19' OR 'Cytokeratin 19' OR 'Ck-19' OR 'Ck19' OR 'Ck 19'):ti,ab,kw | 529 |
| #10 | MeSH descriptor: [Ultrasonography] explode all trees | 17519 |
| #11 | MeSH descriptor: [Ultrasonics] explode all trees | 383 |
| #12 | ('ultrasound*' OR 'echotomography' OR 'sonography' OR 'ultrasonographic' OR 'echography' OR 'ultrasonography' OR 'ultrasonic' OR 'phonophoresis' OR 'sonication' OR 'sonification' OR 'ultra sound' OR 'doptone' OR 'echogram' OR 'echographic' OR 'echoscopy' OR 'echosound' OR 'sonogram' OR 'sonographic' OR 'ultrasonogram'):ti,ab,kw | 57628 |
| #13 | (#1 OR #2) AND (#3 OR #4 OR #5) AND (#6 OR #7) | 32 |
| #14 | (#1 OR #2) AND (#3 OR #4 OR #5) AND (#8 OR #9) | 10 |
| #15 | (#1 OR #2) AND (#10 OR #11 OR #12) AND (#6 OR #7) | 12 |
| #16 | (#1 OR #2) AND (#10 OR #11 OR #12) AND (#8 OR #9) | 1 |

**3.Embase**

| Search number | Query | Results |
| --- | --- | --- |
| #1 | 'liver cell carcinoma'/exp OR 'liver cancer*':ti,ab,kw OR 'liver cell carcinoma*':ti,ab,kw OR 'hepatic carcinoma*':ti,ab,kw OR 'hepatic cell carcinoma*':ti,ab,kw OR 'hepatocarcinoma*':ti,ab,kw OR 'hepatocellular carcinoma*':ti,ab,kw OR 'hepatoma*':ti,ab,kw OR 'liver carcinoma*':ti,ab,kw OR 'liver carcinoma rupture':ti,ab,kw OR 'malignant hepatoma*':ti,ab,kw OR 'primary liver carcinoma*':ti,ab,kw OR 'hcc':ti,ab,kw OR 'hccs':ti,ab,kw OR 'hepatic cancer*':ti,ab,kw OR 'hepatic neoplasia':ti,ab,kw OR 'hepatic neoplasm*':ti,ab,kw OR 'hepatic tumor*':ti,ab,kw OR 'hepatic tumour*':ti,ab,kw OR 'hepatocellular cancer*':ti,ab,kw OR 'hepatocellular neoplasia':ti,ab,kw OR 'hepatocellular neoplasm*':ti,ab,kw OR 'hepatocellular tumor*':ti,ab,kw OR 'hepatocellular tumour*':ti,ab,kw OR 'liver cell tumor*':ti,ab,kw OR 'liver cell tumour*':ti,ab,kw OR 'liver neoplasia':ti,ab,kw OR 'liver neoplasm*':ti,ab,kw OR 'liver tumour*':ti,ab,kw OR 'liver tumor*':ti,ab,kw OR 'hepatic malignancies':ti,ab,kw OR 'hepatic malignancy':ti,ab,kw OR 'hepatocellular malignancies':ti,ab,kw OR 'hepatocellular malignancy':ti,ab,kw OR 'liver cell cancer*':ti,ab,kw OR 'liver malignancies':ti,ab,kw OR 'liver malignancy':ti,ab,kw OR 'malignant hepatic tumor*':ti,ab,kw OR 'malignant hepatic tumour*':ti,ab,kw OR 'malignant liver neoplasm*':ti,ab,kw OR 'malignant liver tumor*':ti,ab,kw OR 'malignant liver tumour*':ti,ab,kw OR 'primary liver cancer*':ti,ab,kw | 302011 |
| #2 | 'radiomics'/exp OR 'nuclear magnetic resonance imaging'/exp OR 'computer assisted tomography'/exp OR 'x-ray computed tomography'/exp OR 'computer assisted tomography':ti,ab,kw OR 'computerized axial tomography':ti,ab,kw OR 'computerised axial tomography':ti,ab,kw OR 'computed axial tomography':ti,ab,kw OR 'cat':ti,ab,kw OR 'computerised tomography':ti,ab,kw OR 'computerized tomography':ti,ab,kw OR 'computed tomography':ti,ab,kw OR 'computer tomography':ti,ab,kw OR 'computed tomographic':ti,ab,kw OR 'ct':ti,ab,kw OR 'nuclear magnetic resonance imaging':ti,ab,kw OR 'magnetic resonance imaging':ti,ab,kw OR 'magnetic resonance image*':ti,ab,kw OR 'mri':ti,ab,kw OR 'mr imaging':ti,ab,kw OR 'nmr imaging':ti,ab,kw OR 'magnetic resonance tomography':ti,ab,kw OR 'mr tomography':ti,ab,kw OR 'nuclear magnetic resonance tomography':ti,ab,kw OR 'nmr tomography':ti,ab,kw OR 'radiomic*':ti,ab,kw OR 'radiogenomic':ti,ab,kw OR 'radiomics-based':ti,ab,kw OR 'texture':ti,ab,kw OR 'machine learning':ti,ab,kw OR 'transfer learning':ti,ab,kw OR 'deep learning':ti,ab,kw OR 'ensemble learning':ti,ab,kw OR 'artificial intelligence':ti,ab,kw OR 'random forest':ti,ab,kw OR 'neural network':ti,ab,kw OR 'neural networks':ti,ab,kw OR 'cnn':ti,ab,kw OR 'support vector machine':ti,ab,kw OR 'svm':ti,ab,kw OR 'gradient boosting machine':ti,ab,kw OR 'nomogram':ti,ab,kw OR 'xgboost':ti,ab,kw OR 'adaboost':ti,ab,kw OR 'decision tree':ti,ab,kw OR 'resnet-50':ti,ab,kw OR 'resnet':ti,ab,kw OR 'naive bayesian':ti,ab,kw OR 'multilayer perceptron':ti,ab,kw OR 'bayesian network':ti,ab,kw OR 'transformer':ti,ab,kw OR 'dnn':ti,ab,kw OR 'rnn':ti,ab,kw | 2988404 |
| #3 | 'ki 67 antigen'/exp OR 'ki 67 antigen':ti,ab,kw OR 'ki-67 antigen':ti,ab,kw OR 'ki-67':ti,ab,kw OR 'ki67':ti,ab,kw OR 'ki 67':ti,ab,kw OR 'mib-1':ti,ab,kw OR 'mib1':ti,ab,kw OR 'mib 1':ti,ab,kw OR 'antigen ki-67':ti,ab,kw OR 'antigen ki 67':ti,ab,kw OR 'mib-1 antigen':ti,ab,kw OR 'mib 1 antigen':ti,ab,kw OR 'antigen ki67':ti,ab,kw | 97909 |
| #4 | 'cytokeratin 19'/exp OR 'keratin 19':ti,ab,kw OR 'keratin-19':ti,ab,kw OR 'cytokeratin-19':ti,ab,kw OR 'cytokeratin 19':ti,ab,kw OR 'ck-19':ti,ab,kw OR 'ck19':ti,ab,kw OR 'ck 19':ti,ab,kw | 11662 |
| #5 | #1 AND #2 AND #3 | 544 |
| #6 | #1 AND #2 AND #4 | 345 |
| #7 | ('echography/exp' OR 'ultrasound/exp' OR 'ultrasound*':ti,ab,kw OR 'echotomography':ti,ab,kw OR 'sonography':ti,ab,kw OR 'ultrasonographic':ti,ab,kw OR 'echography':ti,ab,kw OR 'ultrasonography':ti,ab,kw OR 'ultrasonic':ti,ab,kw OR 'phonophoresis':ti,ab,kw OR 'sonication':ti,ab,kw OR 'sonification':ti,ab,kw OR 'ultra sound':ti,ab,kw OR 'doptone':ti,ab,kw OR 'echogram':ti,ab,kw OR 'echographic':ti,ab,kw OR 'echoscopy':ti,ab,kw OR 'echosound':ti,ab,kw OR 'sonogram':ti,ab,kw OR 'sonographic':ti,ab,kw OR 'ultrasonogram':ti,ab,kw) AND [01-01-1800]/sd NOT [11-05-2023]/sd | 724603 |
| #8 | #1 AND #3 AND #7 | 127 |
| #9 | #1 AND #4 AND #7 | 70 |

**4.Web of science**

| Search number | Query | Results |
| --- | --- | --- |
| 1 | TI=("Hepatocellular Carcinoma*" OR "Liver Cancer*" OR "Liver Cell Carcinoma*" OR "Hepatocellular Carcinoma*" OR "Hepatoma*" OR "hepatic carcinoma*" OR "hepatic cell carcinoma*" OR "hepatocarcinoma*" OR "hepatocellular carcinoma*" OR "hepatoma*" OR "liver carcinoma*" OR "liver carcinoma rupture" OR "malignant hepatoma*" OR "primary liver carcinoma*" OR "HCC" OR "HCCs" OR "hepatic cancer*" OR "hepatic neoplasia" OR "hepatic neoplasm*" OR "hepatic tumor*" OR "hepatic tumour*" OR "hepatocellular cancer*" OR "hepatocellular neoplasia" OR "hepatocellular neoplasm*" OR "hepatocellular tumor*" OR "hepatocellular tumour*" OR "liver cell tumor*" OR "liver cell tumour*" OR "liver neoplasia" OR "liver neoplasm*" OR "liver tumour*" OR "liver tumor*" OR "hepatic malignancies" OR "hepatic malignancy" OR "hepatocellular malignancies" OR "hepatocellular malignancy" OR "liver cell cancer*" OR "liver malignancies" OR "liver malignancy" OR "malignant hepatic tumor*" OR "malignant hepatic tumour*" OR "malignant liver neoplasm*" OR "malignant liver tumor*" OR "malignant liver tumour*" OR "primary liver cancer*") OR AB=("Hepatocellular Carcinoma*" OR "Liver Cancer*" OR "Liver Cell Carcinoma*" OR "Hepatocellular Carcinoma*" OR "Hepatoma*" OR "hepatic carcinoma*" OR "hepatic cell carcinoma*" OR "hepatocarcinoma*" OR "hepatocellular carcinoma*" OR "hepatoma*" OR "liver carcinoma*" OR "liver carcinoma rupture" OR "malignant hepatoma*" OR "primary liver carcinoma*" OR "HCC" OR "HCCs" OR "hepatic cancer*" OR "hepatic neoplasia" OR "hepatic neoplasm*" OR "hepatic tumor*" OR "hepatic tumour*" OR "hepatocellular cancer*" OR "hepatocellular neoplasia" OR "hepatocellular neoplasm*" OR "hepatocellular tumor*" OR "hepatocellular tumour*" OR "liver cell tumor*" OR "liver cell tumour*" OR "liver neoplasia" OR "liver neoplasm*" OR "liver tumour*" OR "liver tumor*" OR "hepatic malignancies" OR "hepatic malignancy" OR "hepatocellular malignancies" OR "hepatocellular malignancy" OR "liver cell cancer*" OR "liver malignancies" OR "liver malignancy" OR "malignant hepatic tumor*" OR "malignant hepatic tumour*" OR "malignant liver neoplasm*" OR "malignant liver tumor*" OR "malignant liver tumour*" OR "primary liver cancer*") | 229633 |
| 2 | TI=("Computer Assisted Tomography" OR "Computerized Axial Tomography" OR "computerised axial tomography" OR "computed axial tomography" OR "CAT" OR "computerised tomography" OR "Computerized Tomography" OR "Computed Tomography" OR "computer tomography" OR "computed tomographic" OR "CT" OR "Magnetic Resonance Imaging" OR "Magnetic Resonance Image*" OR "MRI" OR "mr imaging" OR "nuclear magnetic resonance imaging" OR "NMR imaging" OR "magnetic resonance tomography" OR "MR Tomography" OR "nuclear magnetic resonance Tomography" OR "NMR Tomography" OR "Radiomics" OR "radiomic" OR "radiogenomic" OR "radiomics-based" OR "Texture" OR "machine learning" OR "Transfer Learning" OR "Deep learning" OR "Ensemble Learning" OR "artificial intelligence" OR "random forest" OR "neural network" OR "neural networks" OR "CNN" OR "Support vector machine" OR "SVM" OR "Gradient Boosting Machine" OR "Nomogram" OR "XGBoost" OR "Adaboost" OR "Decision tree" OR "ResNet-50" OR "ResNet" OR "Naive Bayesian" OR "Multilayer perceptron" OR "Bayesian network" OR "Transformer" OR "DNN" OR "RNN") OR AB=("Computer Assisted Tomography" OR "Computerized Axial Tomography" OR "computerised axial tomography" OR "computed axial tomography" OR "CAT" OR "computerised tomography" OR "Computerized Tomography" OR "Computed Tomography" OR "computer tomography" OR "computed tomographic" OR "CT" OR "Magnetic Resonance Imaging" OR "Magnetic Resonance Image*" OR "MRI" OR "mr imaging" OR "nuclear magnetic resonance imaging" OR "NMR imaging" OR "magnetic resonance tomography" OR "MR Tomography" OR "nuclear magnetic resonance Tomography" OR "NMR Tomography" OR "Radiomics" OR "radiomic" OR "radiogenomic" OR "radiomics-based" OR "Texture" OR "machine learning" OR "Transfer Learning" OR "Deep learning" OR "Ensemble Learning" OR "artificial intelligence" OR "random forest" OR "neural network" OR "neural networks" OR "CNN" OR "Support vector machine" OR "SVM" OR "Gradient Boosting Machine" OR "Nomogram" OR "XGBoost" OR "Adaboost" OR "Decision tree" OR "ResNet-50" OR "ResNet" OR "Naive Bayesian" OR "Multilayer perceptron" OR "Bayesian network" OR "Transformer" OR "DNN" OR "RNN") | 2719889 |
| 3 | TI=("Ki 67 Antigen" OR "Ki-67 Antigen" OR "Ki-67" OR "Ki67" OR "Ki 67" OR "MIB-1" OR "MIB1" OR "MIB 1" OR "Antigen Ki-67" OR "Antigen Ki 67" OR "MIB-1 Antigen" OR "MIB 1 Antigen" OR "Antigen Ki67") OR AB=("Ki 67 Antigen" OR "Ki-67 Antigen" OR "Ki-67" OR "Ki67" OR "Ki 67" OR "MIB-1" OR "MIB1" OR "MIB 1" OR "Antigen Ki-67" OR "Antigen Ki 67" OR "MIB-1 Antigen" OR "MIB 1 Antigen" OR "Antigen Ki67") | 50584 |
| 4 | TI=("Keratin 19" OR "Keratin-19" OR "Cytokeratin-19" OR "Cytokeratin 19" OR "Ck-19" OR "Ck19" OR "Ck 19") OR AB=("Keratin 19" OR "Keratin-19" OR "Cytokeratin-19" OR "Cytokeratin 19" OR "Ck-19" OR "Ck19" OR "Ck 19") | 5716 |
| 5 | #1 AND #2 AND #3 | 98 |
| 6 | #1 AND #2 AND #4 | 72 |
| 7 | TI=("Ultrasonic" OR "Ultrasonography" OR "Ultrasound*" OR "Echotomography" OR "Ultrasonic" OR "Sonography" OR "Ultrasonographic" OR "Echography" OR "Ultrasonography" OR "phonophoresis" OR "sonication" OR "sonification" OR "ultra sound" OR "doptone" OR "echography" OR "echogram" OR "echographic" OR "echoscopy" OR "echosound" OR "sonogram" OR "sonographic" OR "sonographic" OR "sonography" OR "ultrasonogram" OR "ultrasonographic" OR "ultrasonography" OR "echography") OR AB=("Ultrasonic" OR "Ultrasonography" OR "Ultrasound*" OR "Echotomography" OR "Ultrasonic" OR "Sonography" OR "Ultrasonographic" OR "Echography" OR "Ultrasonography" OR "phonophoresis" OR "sonication" OR "sonification" OR "ultra sound" OR "doptone" OR "echography" OR "echogram" OR "echographic" OR "echoscopy" OR "echosound" OR "sonogram" OR "sonographic" OR "sonographic" OR "sonography" OR "ultrasonogram" OR "ultrasonographic" OR "ultrasonography" OR "echography") | 635161 |
| 8 | #1 AND #3 AND #7 | 35 |
| 6 | #1 AND #4 AND #7 | 21 |
